# The loss of *DHX15* impairs endothelial energy metabolism, lymphatic drainage and tumor metastasis in mice

**DOI:** 10.1038/s42003-021-02722-w

**Published:** 2021-10-15

**Authors:** Jordi Ribera, Irene Portolés, Bernat Córdoba-Jover, Juan Rodríguez-Vita, Gregori Casals, Bernardino González-de la Presa, Mariona Graupera, Estel Solsona-Vilarrasa, Carmen Garcia-Ruiz, José C. Fernández-Checa, Guadalupe Soria, Raúl Tudela, Anna Esteve-Codina, Guadalupe Espadas, Eduard Sabidó, Wladimiro Jiménez, William C. Sessa, Manuel Morales-Ruiz

**Affiliations:** 1 Biochemistry and Molecular Genetics Department, Hospital Clínic of Barcelona, Institut d’Investigacions Biomèdiques August Pi i Sunyer (IDIBAPS), Centro de Investigación Biomédica en Red de Enfermedades Hepáticas y Digestivas (CIBERehd), Barcelona, Spain; 2 German Cancer Research Center, Heidelberg, Germany; 3 Vascular Signalling Laboratory, Program Against Cancer Therapeutic Resistance (ProCURE), Institut d’Investigació Biomèdica de Bellvitge (IDIBELL). CIBERonc, Barcelona, Spain; 4 Cell Death and Proliferation, Institute of Biomedical Research of Barcelona (IIBB), Consejo Superior Investigaciones Científicas (CSIC), Liver Unit, Hospital Clínic, IDIBAPS, Universitat de Barcelona, Barcelona, 08036 Spain; 5 CIBERehd, Instituto de Salud Carlos III, Madrid, 28029 Spain; 6 USC Research Center for ALPD, Keck School of Medicine, Los Angeles, CA 90033 USA; 7 Experimental 7T-MRI Unit, IDIBAPS, Barcelona, Spain; 8 CIBERbbn, University of Barcelona, Barcelona, Spain; 9 CNAG-CRG, Centre for Genomic Regulation (CRG), Barcelona Institute of Science and Technology (BIST), Universitat Pompeu Fabra (UPF), Barcelona, Spain; 10 Proteomics Unit, Centre for Genomic Regulation (CRG), The Barcelona Institute for Science and Technology, Universitat Pompeu Fabra, Barcelona, Spain; 11 Department of Biomedicine-Biochemistry Unit, School of Medicine University of Barcelona, Barcelona, Spain; 12 Department of Pharmacology, Department of Cardiology, Vascular Biology and Therapeutics Program, Yale University School of Medicine, New Haven, CT USA

**Keywords:** Cell signalling, RNA splicing

## Abstract

DHX15 is a downstream substrate for Akt1, which is involved in key cellular processes affecting vascular biology. Here, we explored the vascular regulatory function of DHX15. Homozygous *DHX15* gene deficiency was lethal in mouse and zebrafish embryos. DHX15^—/—^ zebrafish also showed downregulation of VEGF-C and reduced formation of lymphatic structures during development. *DHX15*^*+/−*^ mice depicted lower vascular density and impaired lymphatic function postnatally. RNAseq and proteome analysis of DHX15 silenced endothelial cells revealed differential expression of genes involved in the metabolism of ATP biosynthesis. The validation of these results demonstrated a lower activity of the Complex I in the mitochondrial membrane of endothelial cells, resulting in lower intracellular ATP production and lower oxygen consumption. After injection of syngeneic LLC1 tumor cells, DHX15^+/−^ mice showed partially inhibited primary tumor growth and reduced lung metastasis. Our results revealed an important role of DHX15 in vascular physiology and pave a new way to explore its potential use as a therapeutical target for metastasis treatment.

## Abstract (web summary)

Jordi Ribera et al. use zebrafish and mouse models to examine the role of the ATP-dependent RNA helicase, DHX15, in vascular health. Their results suggest that DHX15 depletion can cause vascular defects in vertebrates, potentially by impacting endothelial ATP production.

## Introduction

RNA helicases are highly conserved and widespread enzymes that play a fundamental role in RNA metabolism through the control of basic RNA processes such as ribosome formation, pre-mRNA maturation, nuclear transportation, RNA translation, transcription initiation, degradation, and folding of RNA^1^. They also play an essential role in the detection of viral RNAs^2^. RNA helicases are classified into two different subfamilies: the DEAD-box family (DDX), and the DEAH-box (DHX) family^2,3^. Specifically, the DHX family consists of 16 members, which have been identified based on their homology within the amino acid sequences of the helicase domain^3,4^.

RNA helicases act by remodeling RNA structures through ATP hydrolysis, which exerts a mechanical force resulting in the alteration of the RNA configuration that is fundamental for many cellular processes. This RNA reconfiguration is due to the translocation of the helicase along the RNA which unwinds RNA duplexes and dissociates bound proteins^5–7^. It has been recently discovered that since DHX helicases lack target selectivity they require the action of adapter proteins, such as G-patch proteins, to aid in the recruitment of RNA targets to the functional site of the helicase. These kinds of DHX activators stabilize a functional conformation with high RNA affinity enhancing the catalytic activity of the helicase^8^.

In recent years, RNA helicases have gained notoriety due to their role in cell maintenance, controlling many biological processes, including cell differentiation and apoptosis^9^. Also, several groups have linked the defects in helicase functioning with cancer, infectious diseases, immune response, and neurodegenerative disorders^2,10^. For instance, recent studies have shown that the deregulated expression of an increasing number of these enzymes usually appears in many types of tumors^11,12^, hence being related to carcinogenesis and cancer progression^13–17^. Despite these initial studies, the understanding of the pathological mechanisms driven by the RNA helicases and their individual specificity or redundancy over their molecular targets is still limited.

DHX15 is a newly identified member of the DEAH-box RNA helicase family, located in the cell nucleus that regulates pre-mRNA maturation^18,19^. DHX15 is known to contribute to ribosome biogenesis by participating in some steps of the small subunit maturation, and in splicing by dissociating the spliceosome modules after completion of its function^20–23^. DHX15 has ubiquitous variable expression in healthy tissues and organs^24^. This helicase is also present in retinal endothelial cells that line the arborizing microvasculature in the human retina^25^. In pathological situations, some studies have shown that DHX15 expression can be dysregulated due to exacerbated autoimmune response and promotes antiviral defense against RNA viruses as a coreceptor for Nlrp6 or Rig-I^26–28^. Also, DHX15 is downregulated in ulcerative colitis patients, promoting intestinal antibacterial response through Wnt/β-catenin signaling^29^ and its expression is altered in several types of cancer^30–33^.

Among all these physiological and pathophysiological mechanisms found to be associated with DHX15, its role in vascular function is still unknown. In this context, the serine/threonine kinase Akt1 plays an essential role in vascular biology as a central signaling node that coordinates major cellular processes^34–36^. Its primary signaling function relays on the fact that Akt1-dependent phosphorylation leads to the regulation of critical mediators that control different cellular processes, including cell death, cell growth, and chemotaxis. In a previous study, we demonstrated that only the Akt1 isoform can phosphorylate DHX15 in mouse lung endothelial cells^35^. This observation led us to hypothesize that DHX15 contributes to some extent to the vascular functions of Akt. Therefore, the goal of the present study was to characterize the vascular phenotypes and the pathological mechanism associated with the *DHX15* gene deficiency generated by gene editing of this enzyme in mice and zebrafish.

The results of our study revealed a vascular regulatory role for DHX15 that has an impact in pathological processes such as impaired lymphatic drainage and tumor growth. Also, RNAseq and proteome analysis of DHX15 silenced endothelial cells revealed differential expression of genes involved in the metabolism of ATP biosynthesis. The validation of these results demonstrated lower activity of the Complex I in the mitochondrial membrane of endothelial cells, resulting in lower intracellular ATP production and lower oxygen consumption.

## Results

### Akt and DHX15 crosstalk in endothelial cells

**Fig. 1:**
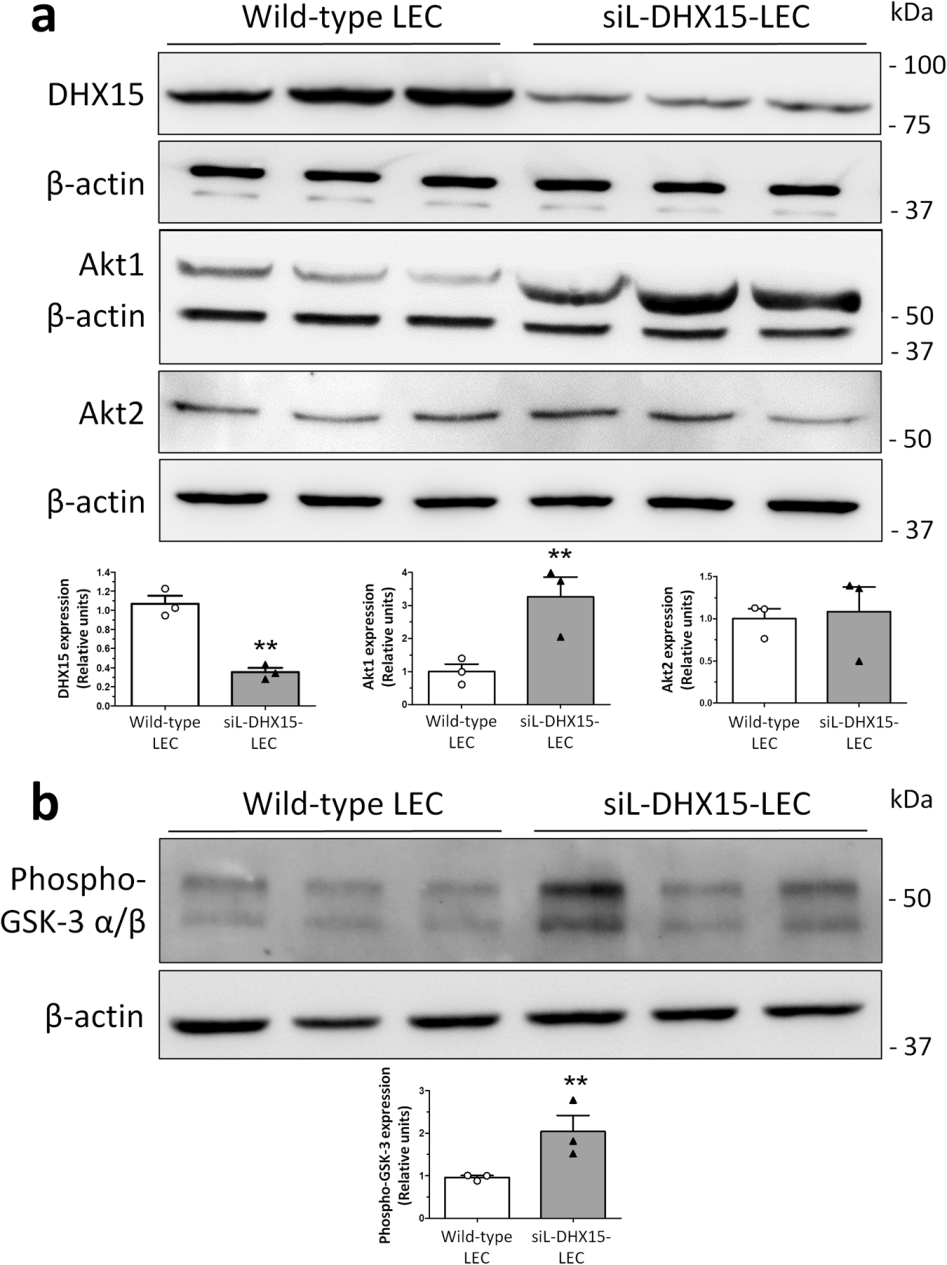
Characterization of the Akt and DHX15 signaling crosstalk in endothelial cells. **a** The expression of DHX15, Akt1, and Akt2 proteins was evaluated by western blot using cell lysates from wild-type and siL-DHX15-LECs. β-actin was used as a loading control. The densitometric analysis of the protein expression is shown on the bar graph. **p < 0.01 vs. wild-type LEC (*n* = 3 biologically independent samples for each condition). **b** The activity of Akt was determined in non-silenced and DHX15-silenced endothelial cells in a kinase reaction using recombinant GSK-3 as substrate. The levels of GSK-3α/β phosphorylation were analyzed by western blot using a phospho-GSK-3 specific antibody. β-actin was used as a loading control. The densitometric analysis of the protein expression is shown on the bar graph. **p < 0.01 vs. wild-type LEC (*n* = 3 biologically independent samples for each condition). All statistical analyses were performed using unpaired two-tailed Student’s *t*-test. All bar graphs are presented as mean ± SEM.

Akt activity is necessary for major endothelial cell functions, including cell growth, chemotaxis, survival, vascular tone, and angiogenesis. We previously demonstrated that DHX15 is a downstream phosphorylation target of Akt1^35^. Here we investigated further the regulatory crosstalk between these two proteins in endothelial cells, using a LEC cell line engineered to express the Tet-On® induction system for silencing DHX15 (siL-DHX15-LEC) (Supplementary Fig. 1a). LEC displayed endothelial cell markers (CD31, eNOS, and uptake of oxidized low-density lipoprotein; Supplementary Fig. 1b and d) but also classical lymphatic cell markers (LYVE-1 and podoplanin; Supplementary Fig. 1c and d). As shown in Fig. 1a, a three-fold upregulation of Akt1 expression was observed in LEC with silenced *DHX15* gene, compared with control LEC (3.26 ± 0.39 vs. 1.00 ± 0.14 relative units, respectively; *p* < 0.01). Akt1 overexpression mediated by DHX15 silencing is isoform specific since we did not observe a variation in Akt2 expression under the same experimental conditions. Furthermore, we determined AKT activity in siL-DHX15-LEC cells measuring GSK-3α/β phosphorylation. We found two-fold increased levels of AKT activity in siL-DHX15-LEC cells compared with WT cells (Fig. 1b).

### Homozygous loss of DHX15 gene is associated with embryonic lethality in mice

The role of DHX15 in vascular function and vascular development is unknown. Therefore, we generated a global knockout mouse for DHX15 as described under the Methods section. Exon 2 of the *DHX15* gene was selected as TALEN target site for knockout mouse production, resulting in two different DHX15-deficient mouse clones: Mouse-ID#35, and Mouse-ID#39. Both clones were used interchangeably in the subsequent experiments without detecting significant differences in the phenotypes or the results obtained (Supplementary Fig. 2a).

**Fig. 2:**
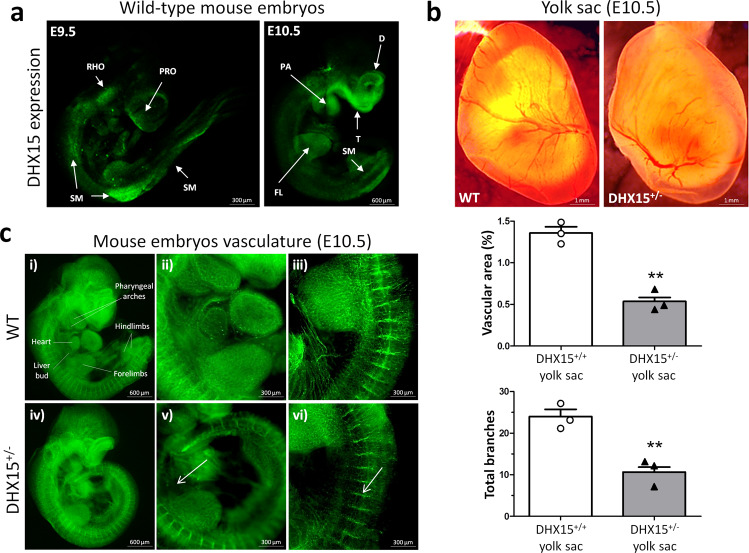
Embryonic characterization of *DHX15* gene deficiency and expression in the gene-edited mouse and zebrafish models. **a** Representative immunostaining of the DHX15 expression (green) from mouse embryos at E9.5 and E10.5 of embryonic development (*n* = 3 animals for each embryonic day). Maximal projection is shown. Original magnification: ×20 for E9.5 and ×10 for E10.5. **b** Representative bright-field images of the yolk sac vasculature from mouse embryos at E10.5 of embryonic development. Maximal projection is shown for each genotype. Original magnification: ×8. The vascular density quantification is shown on the histograms expressed as the percentage of vascular area (upper graph) and total branches (lower graph). Bars represent the mean ± SEM, **p < 0.01 vs. wild-type, unpaired two-tailed Student’s *t*-test (*n* = 3 animals for each condition). **c** Representative immunostaining of the vasculature with endomucin (green) from mouse embryos at the stage E10.5 of embryonic development. The white arrows denote areas of decreased vascular density (*n* = 6 animals for each condition). Maximal projection and 3D rendering from the microscope are shown for each genotype. Original magnification: ×20 and ×40.

DHX15 expression was analyzed in wild-type mouse embryos at embryonic day (E) stage 9.5 and E10.5. The expression of DHX15 in wild-type embryos was mainly located at the prosencephalon (PRO), rhombencephalon (RHO), and somitic mesoderm (SM) at stage E9.5. At stage E10.5, DHX15 expression was still present at the somatic mesoderm, and also was located on the incipient telencephalon (T), diencephalon (D), pharyngeal arches (PA) and forelimbs (FL) (Fig. 2a).

Heterozygous DHX15 (DHX15^+/−^) mice were viable without any apparent phenotypic abnormalities. We confirmed DHX15 heterozygous deficiency in selected organs and vascular beds by RT-qPCR. As seen in Supplementary Fig. 2b we observed a significant reduction of DHX15 expression in kidney, spleen, heart, liver and primary liver endothelial cells from DHX15^+/−^ mice, compared with WT mice. In contrast, intercrosses between DHX15^+/−^ showed no viable homozygous mice (DHX15^−/−^). To establish the embryonic lethality period, timed pregnancies of DHX15^+/−^ breeding were examined at E8.5. No DHX15^−/−^ embryos were obtained at this time point suggesting post-implantation embryonic lethality before E8.5 (Supplementary Fig. 2c).

### *DHX15* gene deficiency causes blood and lymphatic vascular defects during embryonic stages

At stage E10.5, the yolk sac of control mice presented a well-organized vascular network consisting of both capillaries and large vitelline vessels (Fig. 2b). However, DHX15^+/−^ littermates exhibited yolk sacs with reduced vascular density and fewer vascular branches in vitelline vessels, compared with WT (0.54 ± 0.05 *vs*. 1.36 ± 0.07 percentage of vascular area, respectively; *p* < 0.01, and 10.67 ± 1.17 vs. 24.00 ± 1.73 total branches, respectively; *p* < 0.01) (Fig. 2b). Next, we performed whole-mount blood vessel staining in embryos at the same stage, E10.5. DHX15^+/−^ embryos did not exhibit significant defects in segmentation. However, heterozygous embryos showed lower vascular density compared with wild-type (Fig. 2c). This deficiency was mainly evident in the heart region and the intersomitic arteries that sprout out or were located between the somites.

To characterize further the role of DHX15 in vascular definition, we studied whether there was a preferential expression of DHX15 among different vascular beds of wild-type mice. The mRNA quantification of *DHX15* in thoracic aorta and portal vein showed comparable results without significant differences in both vascular territories. Next, we wanted to compare the preferential expression of DHX15 between blood vessels and lymphatic vessels. However, and due to the technical difficulty of isolating entire lymphatic vessels, we quantified DHX15 expression in isolated-primary cells. We found ~3-fold higher expression of DHX15 in primary liver sinusoidal endothelial cells compared with primary mesenteric lymphatic endothelial cells, which revealed a differential expression of DHX15 in the blood vessel endothelium compared with the lymphatic endothelium (Supplementary Fig. 2d). Both cell types, were isolated with high purity and maintained their blood vessel and lymphatic cell markers after the isolation (Supplementary Fig. 3).

**Fig. 3:**
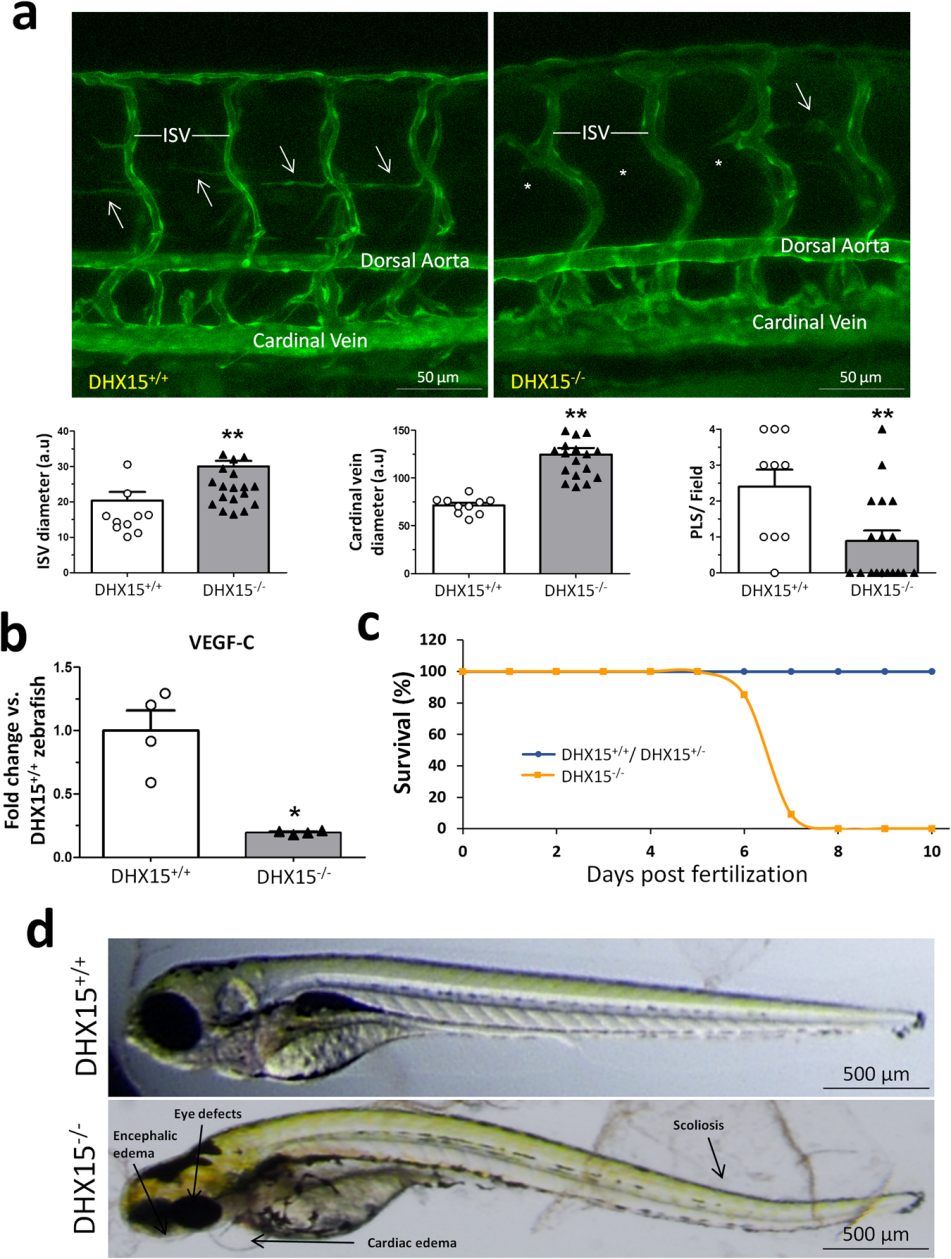
Characterization of embryonic vascular anomalies associated with *DHX15* gene deficient in zebrafish. **a** Representative vascular images of DHX15^+/+^ and DHX15^−/−^ larvae at 5 day post fertilization (dpf) revealing a reduced formation of the parachordal line (arrows). Asterisks denote the absence of these vascular structures in DHX15^−/−^ animals. Quantifications of cardinal vein diameter, intersegmental vessels (ISV), and number of parachordal lymphangioblast strings (PLS) are shown in the graphs. **p < 0.01 vs. wild-type zebrafish, unpaired two-tailed Student’s *t*-test (*n* = 10 and *n* = 18 for the DHX15^+/+^ and DHX15^−/−^ conditions, respectively). Arbitrary units: a.u. **b** RNA extraction of zebrafish embryos at 4 dpf from either wild-type or DHX15 knockout larvae was performed. mRNA expression was analyzed by RT-qPCR. The graph shows the different expression levels of the *VEGF-C* gene in the DHX15^+/+^ and DHX15^−/−^ conditions. mRNA levels are shown as fold change relative to *HPRT* mRNA levels. *p < 0.05 vs. wild-type unpaired two-tailed Student’s *t*-test (*n* = 4 biologically independent samples for each condition). **c** Survival assessment assay. The graph shows the larvae survival rate through the first 10 dpf according to their different genotype (*n* = 15 zebrafish larvae for each condition). **d** Representative images comparing wild-type and DHX15^−/−^ larvae at 7 dpf where morphological defects including encephalic and cardiac edema, scoliosis, and impaired neural/eye growth are evident. All bar graphs are presented as mean ± SEM.

Early embryonic lethality caused in mice by DHX15 deficiency limits the characterization of embryonic vascular anomalies motivated by this genetic disturbance in homozygosis. In order to overcome this limitation, we generated a *DHX15* gene-deficient zebrafish mutant by Crispr/Cas9 editing in a Tg(*flk1*:EGFP) background, as described in the “Methods” section. Wild-type zebrafish embryos showed *DHX15* mRNA expressed broadly across the larvae during the period of 3 days post fertilization (dpf) (Supplementary Fig. 4, panels b, f, and j) with an enriched expression in the vascular system, specifically in the dorsal aorta and the intersegmental vessels (ISV) (Supplementary Fig. 4, panels d, h, and l), as post-natal development progresses (24, 48, and 72 h). DHX15^−/−^ zebrafish embryos at stage 5 dpf were also screened for the expression of GFP in the vasculature. DHX15 deficiency caused vascular development impairment in primary arteries and veins, compared with the wild-type embryos. This impairment was characterized by generalized dilatation of the vasculature, especially in the cardinal vein and the ISV (Fig. 3a). These defects were extended to developing lymphatic structures, such as the parachordal line^37^. In control embryos, the parachordal line was detected in nearly every somite segment (Fig. 3a, arrows). By contrast, the number of parachordal lines was 63% lower in the DHX15^−/−^ embryos. In agreement with the impairment in the formation of embryonic lymphatic structures, DHX15^−/−^ embryos showed downregulation of VEGF-C compared to wild-type embryos (Fig. 3b). These results further support the role of DHX15 as a regulator of the lymphatic endothelium.

Similar to mice, global DHX15 deficiency was lethal in zebrafish. To establish the embryonic lethality period, we monitored the larvae mortality during the first 10 dpf (Fig. 3c). The DHX15^−/−^ larvae started to die at 6 dpf and reached a 100% mortality at the stage 8 dpf. These animals developed morphological defects including encephalic and cardiac edema, scoliosis, and impaired neural/eye growth; compared to wild-type embryos (Fig. 3d), as previously described by McElderry et al.^38^, and the onset of these morphological defects was at 3 dpf. In contrast, DHX15^+/+^ and DHX15^+/−^ did not show significant differences in survival throughout embryonic development or postnatally.

### Vascular density and lymphatic functionality are impaired postnatally in DHX15^+/−^ mice

**Fig. 4:**
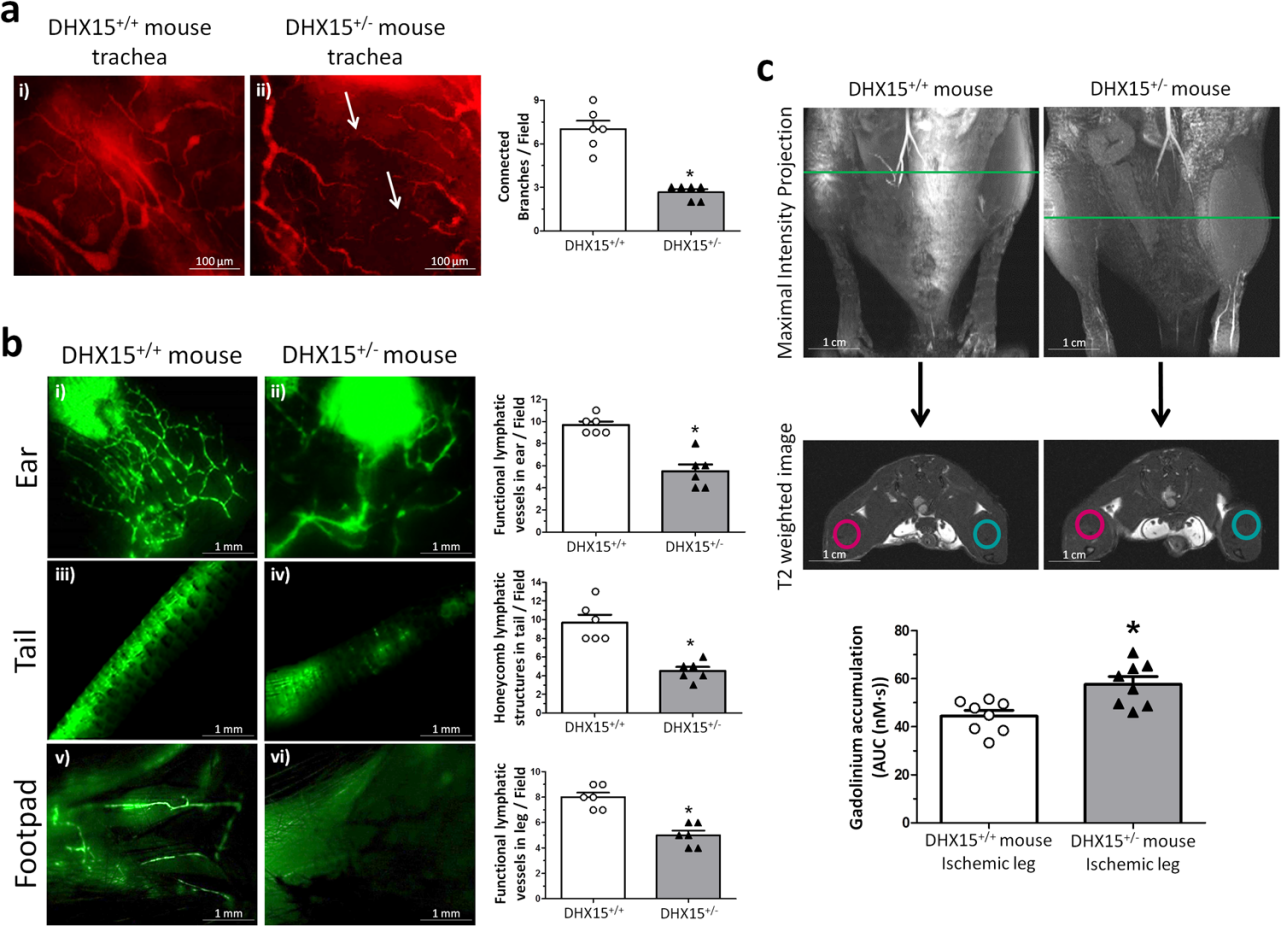
DHX15^+/-^ mice showed cardiovascular and lymphatic vasculature alterations. **a** Representative immunofluorescent images (red CD31 staining) of mouse trachea vessels. White arrowhead evidences lack of connectivity between large vessels. Vascular density quantification is shown on the histogram. *p < 0.05 vs. wild-type (*n* = 5 animals for each contiditon). **b** Lymphatic drainage of 2000 KDa FITC-dextran analyzed by lymphangiography. Fluorescent dye was injected intradermally in the ear (panels i and ii), in the interstitium of the tail-tip (panels iii and iv) and in the footpad (panels v and vi) to assess lymphatic uptake. Lymphatic uptake quantification is shown on the histograms. *p < 0.05 vs. wild-type (*n* = 5 animals for each contidion). **c** Representative magnetic resonance images (MRI) for both strains of mice. First row shows the maximal intensity projection of the time of flight (TOF) angiography. The green line indicates the position of the coronal image (second row: T2-weighted image) where the regions of interest (ROIs) for the analysis of the dynamic contrast enhanced-MRI experiment were positioned. In blue, ROIs for the control leg, in red, ROIs for the ischemic leg. The lower graph shows the area under the concentration curve (AUC) calculated for the ischemic leg in WT and DHX15^+/−^ mice. *p < 0.05 vs. wild-type mouse (n = 8 animals for each condition). All statistical analyses were performed using unpaired two-tailed Student’s *t*-test. All bar graphs are presented as mean ± SEM.

Due to the mortality associated with the lack of DHX15 in homozygotes, it was only possible to characterize the vascular role of DHX15 in viable adult heterozygous mice (DHX15^+/−^). DHX15^+/−^ mice showed significant vascular impairment compared with wild-type mice. Whole-mount preparations of trachea tissue for the quantification of vessel pruning demonstrated that DHX15^+/−^ mice exhibited reduced vascular densities and an impaired connectivity between large vessels (arrows), compared with littermates WT mice (2.67 ± 0.33 vs. 7.00 ± 1.16 connected branches/field, respectively; *p* < 0.05) (Fig. 4a).

We also evaluated lymphatic function in adult DHX15^+/−^ mice by two different methodologies. First, fluorescent lymphangiographies were performed in peripheral regions using FITC-dextran. The lymphatic vasculature of DHX15^+/−^ mice depicted diminished fluid drainage, compared with WT mice in three different peripheral tissues: the ear (5.67 ± 1.20 vs. 9.67 ± 0.33 functional lymphatic vessels/field, respectively; *p* < 0.05), the tail (4.33 ± 0.88 vs. 9.67 ± 1.67 honeycomb lymphatic structures/field, respectively; *p* < 0.05) and the foodpad (5.00 ± 0.63 vs. 8.00 ± 0.63 functional lymphatic vessels/field, respectively; *p* < 0.05) (Fig. 4b). Second, we quantified lymphatic drainage of the contrast agent gadolinium in the hindlimbs after femoral artery ligation. This model is associated with increased vascular permeability and the consequent accumulation of fluid in interstitial spaces. For this purpose, we performed MRI in WT and DHX15^+/−^ mice, 4 weeks after the femoral artery ligation. In the ischemic legs (red circles Fig. 4c), all the DHX15^+/−^ mice showed impaired lymphatic drainage with the consequent significant increase in gadolinium accumulation, as observed by the increased area under the concentration curve calculated in DHX15^+/−^ mice, compared to control animals (57.72 ± 3.19 vs. 44.45 ± 2.35 nM s, respectively; *p* < 0.05).

### Mechanistic insights of DHX15 deficiency in mouse endothelial cells. Role of DHX15 in carbohydrate metabolism

The in vivo experiments showed us that reduced levels of DHX15 caused cardiovascular and lymphatic abnormalities. To obtain information on the mechanisms responsible for these phenotypes in both vascular systems, we performed RNAseq and proteomics in endothelial cells with or without *DHX15* gene silencing. The RNA-seq experiment allowed us to generate 122,968 paired-end reads that were successfully mapped to the mouse reference GRCm38 genome assembly. After quality control analysis, four samples per experimental condition were included in the analysis. A total of 5408 isoforms were differentially expressed when considering a stringent threshold of FDR < 0.001 (Supplementary Data 1). Unsupervised hierarchical clustering analysis of the RNAseq results showed that all the samples from the DHX15-silenced cells clustered together and formed a statistically different group from the wild-type condition, supporting the DHX15 role in the regulation of the endothelial expression program. The heatmap clustering showed in Supplementary Fig. 5a portrays the DHX15-sensitive genes that significantly contribute to the clustering of both experimental conditions.

For the proteomics experiment, protein extracts (*n* = 5 for each experimental condition) were digested into peptides and they were quantified by mass spectrometry analysis, as described in the “Methods” section. We prioritized those protein variations that showed a *p-*value lower than 0.05 or when the protein was present in one condition and undetectable in all the cases of the other experimental condition. The list of proteins with relevant changes in their abundance due to the DHX15 silencing are detailed in Supplementary Data 2.

In an effort to understand the biological relevance of the -omics results, we combined the results of the RNAseq and proteomic experiments and subjected the final list to Gene Set Enrichment based on Gene Ontology Analysis (GSEA-GO) and pathway analysis (IPA; Ingenuity). First, we used the annotations of our differential expressed isoforms to find out which GO terms are changed in response to DHX15 knockdown in endothelial cells (Supplementary Data 3). As seen in Supplementary Fig. 5b, major functions related to vascular growth were underrepresented, such as regulation of chemotaxis, angiogenesis, and wound healing. In contrast, metabolic processes such as ATP biosynthetic process and carbohydrate catabolic process were overrepresented, pointing to these mechanisms as direct targets of DHX15 in endothelial cells. Next, the RNAseq results were compared against global molecular networks to identify associated diseases and canonical pathways affected by the perturbation of DHX15. The DHX15 silencing significantly affects two networks: (1) endocrine system disorders, organismal injury and abnormalities and cancer, and (2) gastrointestinal disease, organismal injury and abnormalities and carbohydrate metabolism (Supplementary Fig. 6a and b). To validate RNAseq results within the glycolytic pathway, we evaluated RNA expression of several participating enzymes such as *pyruvate kinase*, *G3PDH*, *Aldo A* and *UGGT1* (Supplementary Fig. 7). In accordance to RNAseq results, in DHX15-silenced cells, *pyruvate kinase* and *G3PDH* presented a significantly higher expression, whilst analysis in *Aldo A* and *UGGT1* depicted a non-significant tendency towards an increased RNA expression in silenced cells (*p* = 0.49 and *p* = 0.69, respectively).

**Fig. 5:**
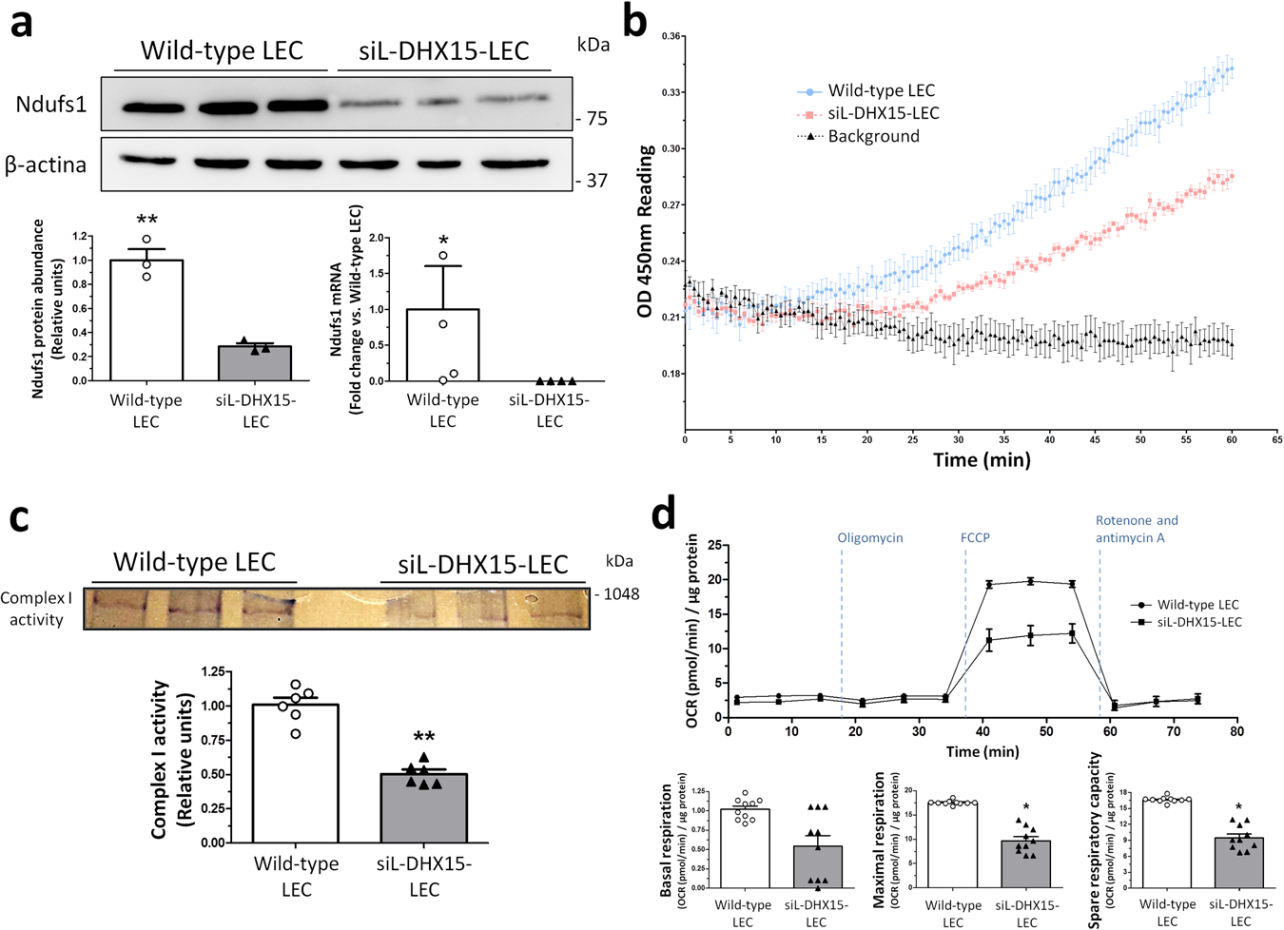
siL-DHX15-LEC showed impaired mitochondrial respiratory chain activity. **a** Protein and gene expression of NDUFS1 was analyzed. Protein was evaluated by western blot using cell lysates from wild-type and siL-DHX15-LEC. β-actin was used as a loading control. The densitometric analysis of the protein expression are shown on the bottom left bar graph. **p < 0.01 (*n* = 3 biologically independent samples for each condition). mRNA expression was analyzed by RT-qPCR. mRNA levels are illustrated as fold change relative to *HPRT* mRNA levels. Bottom right graph. *p < 0.05 (*n* = 4 biologically independent samples for each condition). **b** Complex I activity was assessed using a commercial colorimetric enzymatic reaction reagent for the mitochondrial respiratory complex I in wild-type and siL-DHX15-LEC. Seven hundred µg of total protein was used in each experimental condition (*n* = 3 biologically independent samples for each condition). Wild-type-LEC: upper line, siL-DHX15-LEC: middle line, background: bottom line. **c** *In-gel* activity staining on clear-native page (CN-PAGE) of the respiratory complex I from wild-type and siL-DHX15-LEC’s mitochondria. The quantification of the relative band intensities of complex I activity is shown in the graph below. **p < 0.01 vs. wild-type LEC (*n* = 6 biologically independent samples for each condition). **d** Wild-type and siL-DHX15-LEC were evaluated for basal, coupled and maximal respiration in a mito-stress assay using a Seahorse XFe24 analyzer (*n* = 10 independent Seahorse wells for each condition). OCR is normalized to µg of total protein. *p < 0.05 vs. wild-type LEC. All statistical analyses were performed using unpaired two-tailed Student’s *t*-test. All data are presented as mean ± SEM.

Next, we performed a bioinformatic analysis to determine alternative splicing in genes targeted by DHX15. The rMATS analysis of alternative 5′ splice site from the RNA-seq data identified 220 significant splicing event modifications (with FDR < 5%, absolute difference >5% and >70 number of reads) caused by DHX15 silencing (Supplementary Data 4). Among these genes, NADH ubiquinone oxidoreductase core subunit S1 (*NDUFS1*) showed a differential alternative splicing characterized by the presence of an alternative 5’ splice site, giving rise to a longer exon 1 for NDUFS1, when DHX15 was silenced in LECs (Supplementary Fig. 5c). This gene encodes a subunit of the mitochondrial respiratory chain complex I. To validate these RNA-seq results, NDUFS1 protein expression in LEC cells was analyzed. A significant 70% reduction of NDUFS1 was found in siL-DHX15-LEC (Fig. 5a, bottom graph left). In addition, a reduced NDUFS1 RNA expression was found in siL-DHX15-LEC (Fig. 5a, bottom graph right). To obtain a functional validation of these results, we studied the activity of the mitochondria electron transport chain in endothelial cells. In agreement with the reduced NDUFS1 expression, we found a significant reduction of the complex I activity in siL-DHX15-LEC cells compared with non-silenced LEC cells (Fig. 5b). Such results were confirmed by the in-gel activity measurement, were the activity of complex I was reduced significantly by ~50% in the siL-DHX15-LEC condition, compared with non-silenced LEC cells (0.50 ± 0.04 *vs*. 1.00 ± 0.03 relative units, respectively; *p* < 0.01) (Fig. 5c).

Mitochondrial function was further evaluated with the Seahorse mito-stress test kit, which allows the analysis of mitochondrial function through the measurement of the cellular oxygen consumption rate (OCR). siL-DHX15-LEC depicted a decrease in real-time OCR, leading to reduced rates of basal (0.59 ± 0.33 vs. 1.02 ± 0.11 pmol O_2_/min/µg protein, respectively; *p* = 0.29) and maximal respiration (10.04 ± 2.14 vs. 17.55 ± 0.11 pmol O_2_/min/µg protein, respectively; *p* < 0.05) compared with non-silenced LEC cells. These differences were reflected in a lower spare respiratory capacity of the DHX15 silenced cells (Fig. 5d). Next, we wanted to evaluate the specificity of DHX15 in the regulation of mitochondrial oxygen consumption in endothelial cells by measuring this parameter in hepatocytes. The mito-stress experiments performed in an immortalized hepatocyte cell line under the wild-type and the DHX15 silenced conditions showed no differences in the values of OCR (Supplementary Fig. 8), thus ruling out that the effect of DHX15 on mitochondrial oxygen consumption is an universal mechanism.

**Fig. 6:**
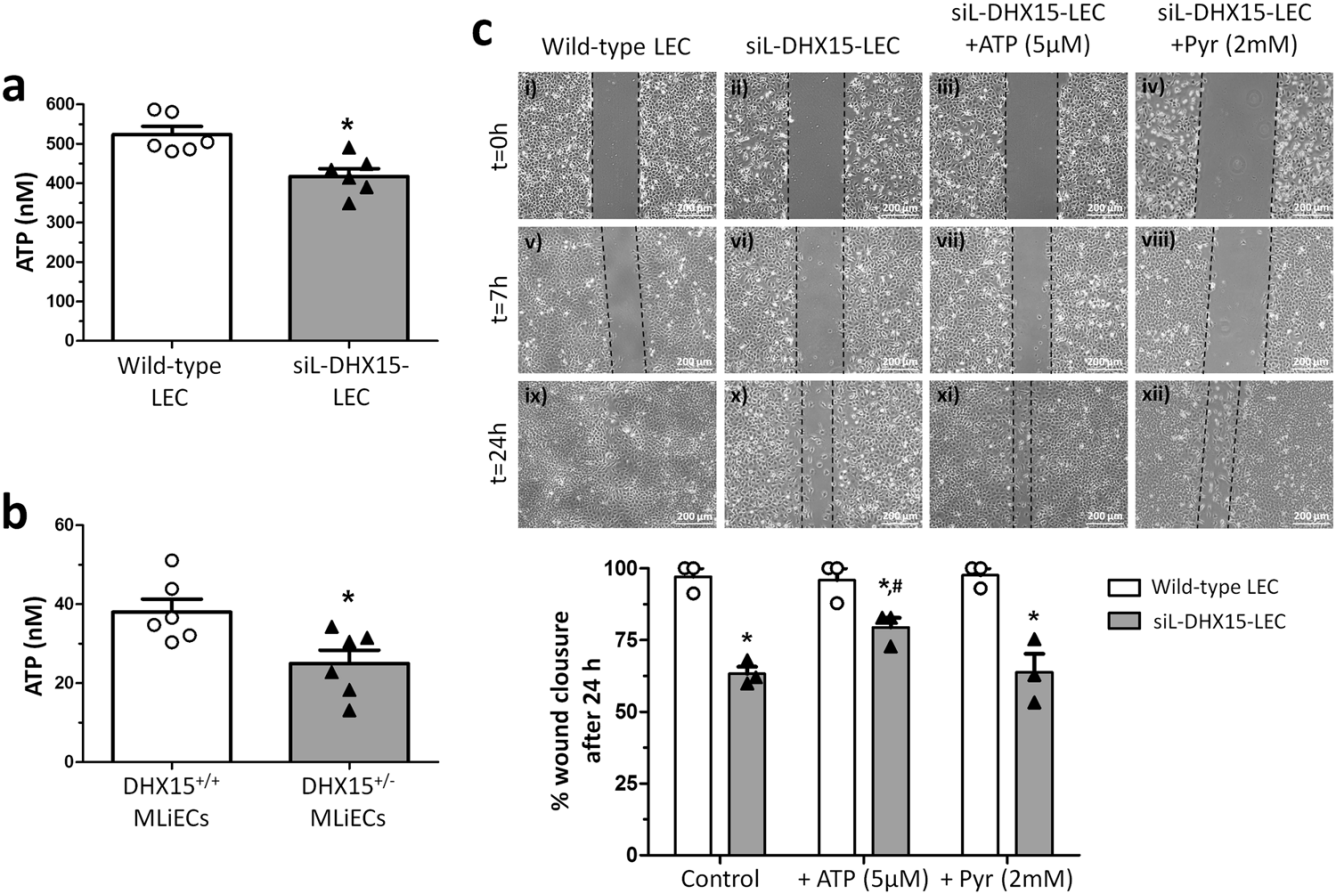
siL-DHX15-LEC presented less cell migration. **a** ATP production was evaluated by a luminescence assay in wild-type and siL-DHX15-LEC. Bars represent the mean ± SEM, *p < 0.05 vs. wild-type LEC (*n* = 6 biologically independent samples for each condition). **b** ATP production was evaluated by a luminescence assay in MLiEC isolated from wild-type and DHX15^+/−^ mice. Bars represent the mean ± SEM, *p < 0.05 vs. wild-type LEC (*n* = 3 biologically independent samples for each condition). **c** Cell migration was quantified after performing a scratch wound in confluent non-silenced and silenced LECs cells that were cultured in six-well plates. Then images of wound healing were acquired after 0, 7, and 24 h (*n* = 6 independent experiments). Graph shows the quantification of the wound closure after 24 h as percentage of migration. Bars represent the mean ± SEM, *p < 0.05 *vs*. respective wild-types and ^*#*^*p* < 0.05 vs. siL-DHX15-LECs without ATP and the pyruvate condition (Pyr). For **a** and **b** statistical analyses were performed using unpaired two-tailed Student’s *t*-test; for **c** statistical analysis was performed using one-way ANOVA with Tukey’s post hoc test for multiple comparisons. All bar graphs are presented as mean ± SEM.

To further confirm the mitochondrial impairment observed in siL-DHX15-LEC, we measured ATP generation in LEC and primary liver endothelial cells. The alterations in mitochondrial activity were linked to lower ATP production compared to WT LEC (417.7 ± 31.37 vs. 524.2 ± 20.40 nM ATP, respectively; *p* < 0.05) (Fig. 6a). Such results were reproduced in primary liver endothelial cells where a lower ATP production was also observed compared to WT cells (24.97 ± 3.41 vs. 38.05 ± 3.23 nM ATP, respectively; *p* < 0.05) (Fig. 6b).

Next, we quantified cell proliferation and migration to assess the impact of reduced energy biosynthesis in siL-DHX15-LEC. Silencing of DHX15 reduced BrdU uptake compared to control LEC (55.53 ± 0.49 vs. 66.43 ± 0.46% of cells BrdU positives, respectively; *p* < 0.01) (Supplementary Fig. 9a). Accordingly, the reduction of DHX15 expression also resulted in an impaired cell migration. In this context, the siL-DHX15-LEC cell line showed delayed wound healing capability after 24 h compared with control LEC (Fig. 6c, panels i, ii, ix, and x).

Next, we evaluated the effect of ATP supplementation over the impaired wound healing observed in siL-DHX15-LEC. With this purpose, we added two additional experimental conditions to the wound healing cell migration experiment: 1/cell culture medium supplemented with 5 µM ATP to equilibrate the lower ATP production of siL-DHX15-LEC, and 2/cell culture medium supplemented with 2 mM pyruvate. The purpose of the last condition was to discard a deficient production of pyruvate that may compromise the levels of acetyl-CoA, and as a result endogenous ATP production, in the mitochondria. The results of this experimental setting showed that ATP (Fig. 6c, panels iii, vii, and xi) but not pyruvate supplementation (Fig. 6c, panels iv, viii, and xii) was able to restore the wound healing capability of siL-DHX15-LEC after 24 h of incubation.

In order to check if some of the results obtained in LEC cells could be extrapolated to the lymphatic vasculature, we performed another scratch-induced wound-healing assay in primary lymphatic endothelial cells. The results were similar that those obtained in LEC cells, LyECs from DHX15^+/−^ presented delayed wound-healing after 24 h compared with control LyECs from DHX15^+/+^ (Supplementary Fig. 9b), indicating that some deficiencies observed in the blood vasculature extrapolated to those of the lymphatic vasculature.

### Heterozygous *DHX15* gene deficiency reduced tumor growth and metastases

Abnormal function of blood and lymphatic vessels plays a pathological role in multiple pathological conditions including inflammation and cancer. Also, there is a strong link between the lymphatic vasculature and tumor spread, as lymphatic vessels constitute one of the main routes of metastasis in most cancers^39–41^. Considering also that one of the pathways modified by the reduction of DHX15 was “endocrine system disorders, organismal injury and abnormalities and cancer”, we aimed at evaluating the role of DHX15 in tumor development.

**Fig. 7:**
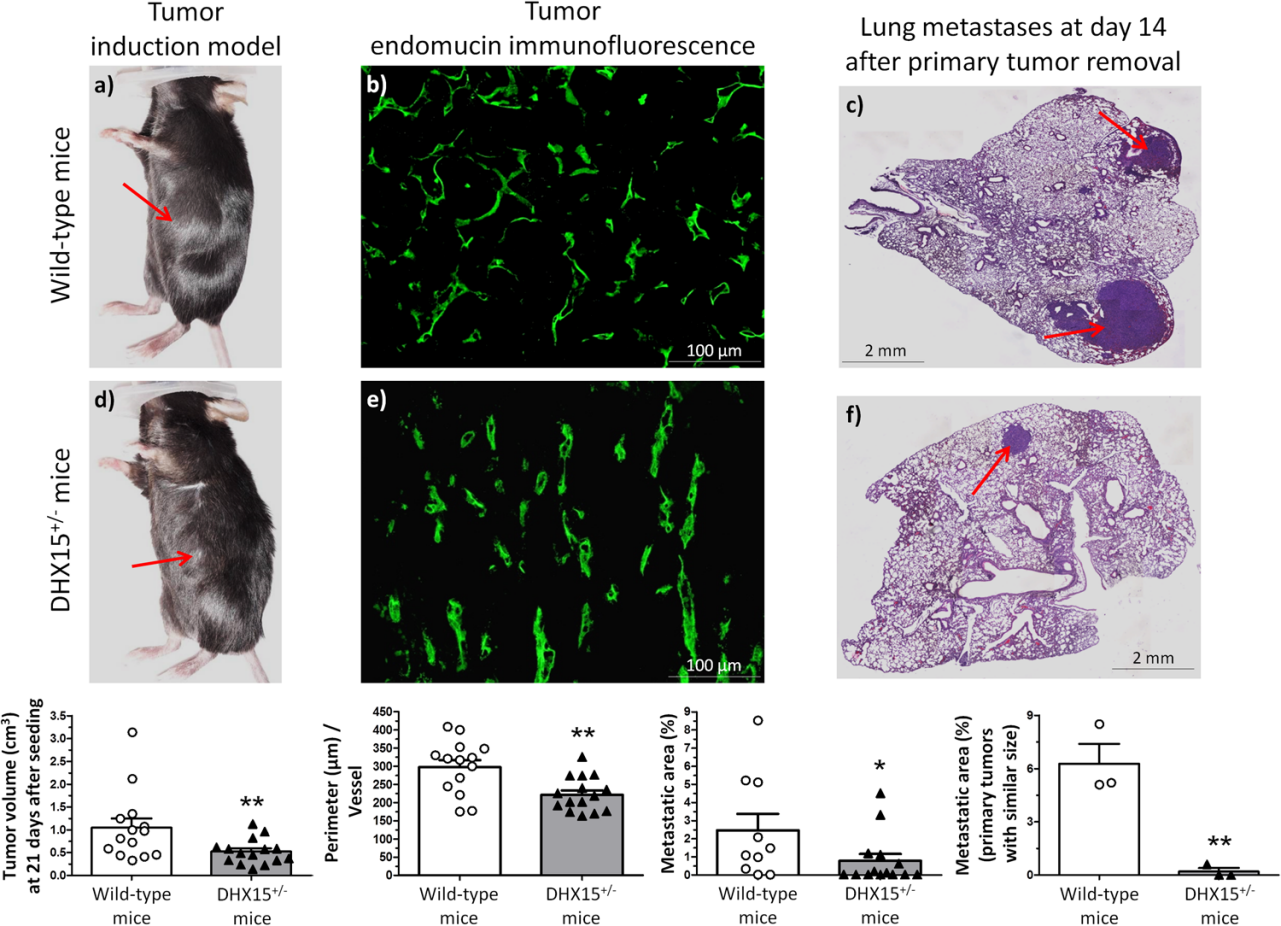
Tumor growth and metastases in DHX15^+/−^ mouse. Macroscopic images of tumor size in wild-type (**a**) and DHX15^+/-^ mice (**d**) three weeks after mouse Lewis lung cancer cells (LLC1) implantation. The arrows indicate the primary tumor. The quantification of tumor volume (cm^3^) is shown on the lower graph. **p < 0.01 vs. wild-type mice (*n* = 15 animals for each condition). Middle panels show endomucin immunostaining of intratumoral blood vessels in wild-type (**b**) and DHX15^+/−^ mice (**e**). The quantification of the total vascular perimeter of all the intratumoral blood vessels that were positive for endomucin immunostaining was performed with the software Image J. Then, the total vascular perimeter per field was divided by the total number of endomucin^+^ vessels per field. The statistical comparison between experimental groups was made considering the result of this index (Perimeter/Vessel). **p < 0.01 vs. wild-type mice (*n* = 15 biologically independent samples for each condition; original magnification: ×200). Right panels show representative lung sections of lung metastatic area after haematoxylin-eosin staining (H&E) in wild-type (**c**) and DHX15^+/−^ mice (**f**). The arrows indicate the metastatic areas. Quantifications of the percentage of lung metastases are shown in the graphs below. In the graph on the left: all tumors. *p < 0.05 vs. wild-type mice (*n* = 15 biologically independent samples for each condition). In the graph on the right: primary tumors with similar size. **p < 0.01 *vs*. wild-type mice (*n* = 3 biologically independent samples for each condition). Original magnification: ×10. All statistical analyses were performed using unpaired two-tailed Student’s *t*-test. All bar graphs are presented as mean ± SEM.

First, we explored how the loss of DHX15 affected the growth of tumors implanted into WT and DHX15^+/−^ mice by injecting syngeneic LLC1 cells (1 × 10^5^) into the flanks of both strains. We measured the volume of the primary tumors implanted in these mice 21 days post-injection of the LLC1 cells. Primary tumors were significantly smaller in DHX15^+/−^ mice compared with controls (0.54 ± 0.07 vs. 1.06 ± 0.17 cm^3^, respectively; *p* < 0.01) (Fig. 7, panels a and d). Tumors from DHX15^+/−^ mice also showed an impaired vascular network characterized by smaller vascular perimeter, compared with WT mice (199.1 ± 9.81 vs. 312.5 ± 17.16 µm perimeter, respectively; *p* < 0.01) (Fig. 7, panels b and e).

Since growth of the primary tumor is rate-limiting and precludes analyses of metastasis in this model, we established a postsurgical metastasis model. primary tumors were completely resected after three weeks of subcutaneous LLC1 cells implantation and postsurgical lung metastasis were evaluated 2 weeks later. Fewer primary tumors from DHX15^+/−^ mice metastasized the lungs compared with wild-type mice (*p* < 0.01) (Supplementary Fig. 10a). Noteworthy, and considering the mice with lung metastasis, the overall area of metastases was strongly decreased in DHX15^+/−^ mice, compared with the wild-type group (0.88 ± 0.32 vs. 2.53 ± 0.69% lung metastases, respectively; *p* < 0.05) (Fig. 7, panels c and f).

To investigate further whether the differences in the size of lung metastasis were due to a potential lower LLC1 cell seeding in the primary tumors, we followed over time a group of mice with similar primary large-size tumors. In this experimental group (size of primary tumors ranging from 0.81 to 1.13 cm^3^), we also found significant differences when we compared the areas of lung metastasis between wild-type and DHX15^+/−^ mice, being significantly lower in the DHX15^+/−^ mice (6.27 ± 1.12 vs. 0.20 ± 0.20% lung metastases, respectively; *p* < 0.01) (Fig. 7, lower right graph). Mice survival was monitored during the experimental procedure. Supplementary Fig. 10b shows that DHX15^+/−^ mice presented a tendency for a higher survival-rate compared with wild-type mice, without reaching statistical significance (83.33 vs. 66.67 percentage of survival at 5 weeks after LLC1 cells implantation, respectively).

To understand the mechanisms that drive the changes in the intratumoral vasculature and the metastatic risk, we next studied the differential expression of molecular regulators in the primary tumors induced by LLC1 allotransplantation. With this purpose, we quantified the intratumoral expression of several angiogenic, lymphangiogenic, and metastasis-related chemokines by RT-qPCR: VEGF-A, VEGF-C, VEGF-D, angiopoietin-1, angiopoietin-2, and SDF-1. Among them, only VEGF-C and stromal cell-derived factor-1 (SDF-1) showed significant changes in the DHX15^+/−^ mice being reduced in this experimental condition compared with wild-type mice (Supplementary Fig. 10c). It is noteworthy that VEGF-C was also downregulated in the DHX15^−/−^ zebrafish experimental model (Fig. 3b). This concordant result points out a conserved regulation of this lymphangiogenic factor by DHX15 across species. Angiopoietin-2 expression in DHX15^+/−^ mice was also decreased compared with the wild-type condition, although this difference did not reach statistical significance (Supplementary Fig. 10c). In addition, VEGF-C downregulation was associated with a significant decrease in intratumoral Lyve-1 immunostaining, which is a specific marker for the presence of lymphatic endothelium (Supplementary Fig. 10d).

## Discussion

In previous studies, we identified DHX15 as a downstream target of Akt1^35^, which has the greatest influence on vascular regulation compared with the other Akt isoforms. For instance, Akt1^-/-^ mouse shows a reduction in postnatal vascular density in different areas such as the myocardium^42^ and the retina^35^. Also, Akt1 activity is critically required during lymphatic development in mice^43^. In the current study, we demonstrated increased AKT1 expression in DHX15 silenced endothelial cells, pointing out the existence of isoform-specific crosstalk between AKT1 and DHX15. All together, let us hypothesize that DHX15 may play a relevant role in vascular function and growth.

Homozygous DHX15 deficiency was associated with embryonic mortality in mice. In contrast, the loss of just one DHX15 allele resulted in viable embryos. However, DHX15^+/−^ mice showed impaired lymphatic drainage and decreased vascular density in the yolk sac and different territories of embryos and adult mice. Importantly, DHX15 protein expression was detected in wild-type mice at the embryonic stages E9.5 and E10.5, which is a key timing for vasculature development and definition^44^.

These in vivo results are in agreement with the reduced cell proliferation and migration that we observed in LEC after silencing the expression of DHX15. Why vasculature is affected by the partial loss of DHX15 in adult mice remains unclear if we only consider the results of this experimental model. Under this experimental setting, we cannot discard the possibility that adult mice developed these vascular defects because of accumulated damage occurring during the postnatal stage in the DHX15 deficiency background. However, and by studying the role of this RNA helicase in *DHX15* gene-deficient zebrafish, we showed that the DHX15-related vascular defects are also occurring during development. In this experimental model, we found that DHX15^−/−^ larvae were not viable. Also, these unviable larvae presented blood vascular alterations and reduced formation of developing lymphatic structures, which were associated with cardiac and encephalic edema in the embryos.

Vascular growth and lymphangiogenesis are crucial in tumor growth and metastasis^45,46^. We found that heterozygous DHX15 deficiency partially inhibits primary tumor growth and reduced lung metastases. This effect was associated with an intratumoral reduction in the length of the blood vessel capillaries. Our results agree with several clinical studies that underline a role of DHX15 in tumor progression in several types of cancer, such as acute myeloid leukemia^33^, hepatocellular carcinoma^47^, malignant peripheral nerve sheath tumors^31^, prostate cancer^48^ and non-small-cell lung cancer^49^. In all these situations, modifications of DHX15 activity and/or its overexpression favors tumor growth. Several mechanisms have been proposed in these studies to explain the tumorigenicity effect of DHX15, such as the co-activation of the androgen receptor in prostate cancer and the transcriptional activation of NF-kB in leukemia cells. In our study, we provided an additional mechanism of DHX15 that is relevant for tumor progression: the regulation of vascular growth and function.

The use of RNAseq and proteomics, combined with bioinformatics down-stream analysis, is one of the most potent approaches to investigate the role played by RNA helicases, as changes in differential splicing of a gene may or may not be associated with changes in its protein abundance; therefore, we need the combined result of both high-throughput approaches. Adopting this strategy, we were able to identify in the DHX15-silenced LEC significant changes in key pathways that metabolize carbohydrates. Some of the gene products that varied were glyceraldehyde-3-phosphate dehydrogenase (*G3PDH*) and pyruvate kinase (*PKM*). These differential expressions were also associated with a reduction in the intracellular concentration of ATP. The upregulation of glycolytic enzymes suggests a compensatory mechanism to counteract the impaired energy production in DHX15 deficient endothelial cells. Supporting this possibility, we observed by RNAseq significant expression changes in members of the family of the NADH dehydrogenase [ubiquinone] 1 alpha subcomplex assembly factor (members from NDUFS1, NDUFS5 and NDUFS7). These members are accessory subunits of the mitochondrial membrane respiratory chain NADH dehydrogenase (Complex I), that transfer electrons from NADH to ubiquinone in the mitochondrial respiratory chain. Among them, we observed a significant alteration in the splicing of the *NDUFS1* gene. This alteration in gene splicing was linked with a 50% reduction of the activity of the complex I in the mitochondria of DHX15 silenced cells that may explain the decrease of ATP intracellular levels and, as a consequence, the impairment in endothelial cell proliferation and vascular growth.

Endothelial cells are mainly considered a glycolytic cell type that maintains their energy demands by exploiting the glycolytic pathway preferentially without the need of coupling to the tricarboxylic acid cycle and the oxidative phosphorylation^50,51^. However, some studies support the notion that mitochondria electron transport chain activity is also playing a role in driving endothelium angiogenesis and function. For example, low concentration of oxygen results in the generation of mitochondrial ROS through the Complex III of the electron transport chain^52^. This increase in mitochondrial ROS regulates angiogenic factors such as HIF-1a, promoting its stabilization and allowing the transcription of the VEGF-A gene^53,54^. Also, HUVEC and HDMEC depend on mitochondrial oxidative phosphorylation to maintain energy supplies for proliferation and growth, as demonstrated by measuring oxygen consumption and ATP production in the presence of the mitochondrial uncoupler embelin^55^. Another transport electron chain inhibitor, rotenone that blocks Complex I activity, reduced the angiogenic capacity of vasa vasorum endothelial cells^56^. Our study is in line with these observations and supports the idea that for optimal angiogenic response, endothelial cells require dynamic crosstalk between glycolysis and mitochondria activity, driven likely by regulators that promote a metabolic switch, as we have seen when we modified the levels of DHX15. Interestingly, DHX15 deficiency impaired mitochondrial oxygen consumption in endothelial cells but not in hepatocytes, thus ruling out that the effect of DHX15 on mitochondrial oxygen consumption is an universal mechanism. We also described other DHX15-related mechanisms independent of the mitochondrial respiratory chain that target the vasculature. In this context, we observed a consistent downregulation of VEGF-C in mouse and zebrafish DHX15-deficient animals, and reduced intratumoral levels of SDF-1 in DHX15^+/−^ mice. VEGF-C and SDF-1 play relevant roles in lymphangiogenesis, metastasis and recruitment of endothelial progenitor cells^57–60^. Therefore, several factors targeted by DHX15 appear to be involved in the control of vascular homeostasis.

The fact that our study described many alterations in the vasculature does not mean that we attribute exclusively the pathophysiological role of DHX15 to endothelial cells. For instance, McElderry et al.^38^ generated the first vertebrate knockout model for DHX15 in zebrafish and described head and embryonic development defects in stages previous to the formation of the cardiovascular system, leading to mortality at a similar embryonic stage as the one described in our DHX15^−/−^ zebrafish model. Similar results have recently been shown using the same experimental model by Cai et al.^61^, introducing an additional role for DHX15 in the hematopoiesis process through the unfolded protein response pathway. We also showed that E9.5-10.5 mice embryos robustly expressed DHX15 in the brain. Therefore, we cannot exclude the possibility that defects in the vasculature of DHX15^+/−^ mouse embryos or DHX15^−/−^ zebrafish embryos may be secondary to the global DHX15 deficiency background. However, we demonstrated in our study the existence of a direct pathological mechanism of DHX15 deficiency on blood endothelial and lymphatic endothelial cells, supporting the direct role of DHX15 in vascular regulation.

## Methods

### DHX15 transgenic mice

*DHX15* gene-deficient C57BL/6 mice were generated by genomic editing by microinjecting TALEN (transcription activator-like effector nuclease technology) RNA in pronucleated oocytes (Cyagen). The mouse *DHX15* gene (GenBank accession number: NM_007839.3; Ensembl: ENSMUSG00000029169) is located on chromosome 5. Fourteen exons have been identified for this gene, with the ATG start codon in exon 1 and the TGA stop codon in exon 14. Exon 2 was selected as TALEN target sites. TALENs were constructed using the Golden Gate Assembly method^62^ and confirmed by sequencing. The amplicons were then purified and sent for DNA sequencing analysis. TALEN mRNAs generated by in vitro transcription were injected into fertilized eggs for knockout mouse production (cDNA sequence: TGTTGGTGAGACTGGGTC). The pups were genotyped by PCR followed by sequence analysis. The positive founders were breeding to the next generation, which was genotyped by PCR and DNA sequencing analysis. DNA sequencing revealed two different DHX15-deficient mouse clones: Mouse-ID#35 that was missing eight bases in one strand, and Mouse-ID#39 that was missing one base in one strand. Wild-type DNA was used as a negative control for sequencing in parallel. The mRNA transcribed from the targeted allele with frameshift undergoes nonsense-mediated decay (NMD).

DHX15-deficient and WT male and female mice were used at 8–12 weeks of age for all the experiments. All animals were kept under constant temperature and humidity in a 12 h controlled dark/light cycle, and they were fed ad libitum on a standard pellet diet. All experimental procedures performed in the models of mouse and zebrafish were approved by the Investigation and Ethics Committees of the Hospital Clínic and the University of Barcelona.

### Mouse genotyping

Mouse genomic DNA was isolated from tail biopsies using a specific kit (Extract-N-Amp™ Tissue PCR Kit; Sigma-Aldrich, Darmstadt, Germany). PCR was performed using the primer pairs to amplify the *DHX15* gene (primer forward: 5′-CACCAACCTGCCCCATACTCCT-3′ and primer reverse: 5′-TGTATTGTCCCAGGGTAAAATGTGTTG-3′). PCR conditions were as follows: 35 cycles at 94 °C for 30 s, 59.3 °C for 30 s, and 72 °C for 60 s. PCR product was sequenced by sanger sequencing to distinguish the *DHX15* wild-type mice and *DHX15* transgenic mice.

### Immunological staining of mouse embryo whole-mounts

Embryos were harvested at different points between E8.5 to E10.5. Embryos at E10.5 were fixed in 4% paraformaldehyde overnight at 4 °C. For immunostaining of whole-mount embryos, after paraformaldehyde fixation, the embryos were sequentially dehydrated in methanol and then incubated in the permeabilization buffer (PBlec) (PBS pH6.8, 1% Tween 20, 1 mM CaCl_2_, 1 mM MgCl_2_, 0.1 mM MnCl_2_) for 20 min at room temperature. After permeabilization, the embryos were incubated with primary antibody rat anti-endomucin or rabbit anti-DHX15 (Abcam, Cambridge, UK) (1:20 dilution) in PBlec buffer overnight at 4 °C. To remove residual primary antibody, embryos were washed with PBT (PBS pH 6.8, 0.1% Tween) for 5 × 10 min. Next, the embryos were incubated with secondary antibody Alexa Fluor 488-conjugated goat anti-rat or Alexa Fluor 488-conjugated goat anti-rabbit (Thermo Fisher, Waltham, MA, USA) (1∶500 dilution) in the dark overnight at 4 °C, and then washed with PBT for 3 × 10 min and postfix in 4% paraformaldehyde. Negative controls for endomucin and DHX15 detection were performed by omitting the primary antibodies in the immunofluorescence reactions. Images were acquired using fluorescence stereomicroscope (Leica Microsystems, Heerbrugg, Switzerland) and immunofluorescence microscope (Nikon Eclipse E600, Kanagawa, Japan) systems.

### Generation of the Zebrafish animal model

Adults wild-type zebrafish (*Danio rerio*), in a Tg(*flk1*:EGFP);Tg(*fabp10*:RFP) background, purchased from KIT -European Zebrafish Resource Center (EZRC), were maintained at 28–29 °C on a light cycle of 14 h light/10 h dark. The Crispr/cas9 design for gene knock-out was performed as follows: Gene sequences were retrieved from http://www.ncbi.nlm.nih.gov/gene and http://www.ensembl.org/Danio_rerio/Info/Index. The sgRNA was designed using the online tool http://crispor.tefor.net, based on exon site and high efficacy and off-target published algorithms. Microinjection was performed at 1-cell stage embryos. Fertilized zebrafish embryos were collected in E3 medium. Then, embryos were grown at 28.5 °C.

### Zebrafish whole-mount in situ hybridization (ISH)

cDNAs were amplified by PCR from a custom zebrafish cDNAs library obtained by RT-PCR from an mRNA pool coming from 5 days post fertilization zebrafish larvae. We included a SP6 sequence linker in reverse primers to directly use the synthesized PCR products as templates to amplify the reverse riboprobe to be used for ISH. For the ISH, embryos were fixed overnight with 4% PFA and washed twice with PBT. Then, embryos were incubated with hybridation buffer (50% formamide, 5× SSC buffer pH 6 (0.75 M NaCl, 0.075 M sodium citrate), 0.1% triton, 50 µg/mL yeast RNA, 50 µg/mL heparin) at least 1 h. Next, embryos were incubated with hybridation buffer containing the reverse riboprobe overnight. Finally, embryos were washed with washing solution (50% formamide, 1× SSC buffer, 0.1% Tween) 30 min twice and with MABT (100 mM maleic acid pH 7.5, 150 mM NaCl, 0.1% Tween) 10 min five times. Stained embryos were processed for imaging with bright field stereoscope to determine the overall expression pattern.

### Zebrafish vascular characterization

Five-day-old larvae obtained by pairwise mating of adult Tg(*flk1*:EGFP;*fab10*:RFP; *DHX15*^*+/−*^) were sorted in two groups depending on two criteria: (a) curly larvae with abnormal development or (b) normal developed larvae. Larvae were flat-mounted and analyzed by confocal imaging (Zeiss AxioObserver Z1) to evaluate putative phenotypical defects in the trunk angiogenesis caused by the gene knockout. Genomic DNA extracted from the whole embryos (using Extract-N-Amp™ Tissue PCR Kit, Sigma) was used for the genotyping after vasculature imaging.

### Mouse femoral artery ligation model and magnetic resonance imaging (MRI)

Mice were anesthetized with a mixture of 4% isoflurane and 100% oxygen. The femoral artery was isolated, and 5-0 suture was tied tightly around artery at a ∼3 mm distance to the inguinal ligament. Mice were allowed 4 weeks to recover following the surgical procedure.

MRI experiments were conducted on a 7T BioSpec 70/30 horizontal animal scanner (Bruker BioSpin, Ettlingen, Germany), equipped with a 12 cm inner diameter actively shielded gradient system (400 mT/m) using a surface coil dedicated to abdominal imaging. Animals were first anesthetized (1.5% isoflurane in a mixture of 30% O_2_ and 70% CO_2_) and the tail vein was cannulated for administration of contrast agent. Then, animals were transferred under the same anesthesia regime to a Plexiglas holder in supine position with a nose cone for administering anesthetic gases and fixed by a tooth bar, ear bars, and adhesive tape. 3D-localizer scans were used to ensure accurate position of the animal’s midline at the level of the posterior limbs in the isocenter of the magnet. T2-weighted images were acquired by a RARE (rapid acquisition with relaxation enhancement) sequence with an effective echo time (TE) of 24 ms, repetition time (TR) 1201 ms and RARE factor 8. Matrix size was 256 × 256 with an in-plane voxel size of 0.156 × 0.156 mm^2^, 15 slices, slice thickness 1 mm, resulting in a field of view (FOV) of 40 × 40 × 15 mm^3^. Time of flight 3D angiography was acquired a FLASH (Fast Low Angle Shot) protocol with TE = 2.4 ms, TR = 14,000 ms, flip angle 20°, 3 averages, matrix size: 448 × 256 × 128, voxel size 0.078 × 0.078 × 0.234 mm³, resulting in a FOV of 35 × 20 × 30 mm³. The shortening of the T1-relaxation time by the contrast agent enhanced the tissue signal. T1 map was acquired using RARE-VTR (rapid acquisition with relaxation enhancement and variable repetition time) sequence, with TE = 7 ms, 6 TR = 200, 400, 800, 1500, 3000, 5500 ms, matrix size: 96 × 96 × 3, voxel size 0.417 × 0.417 × 1 mm³, resulting in a FOV of 40 × 40 × 3 mm³.

To estimate the T1-relaxation rate and to measure the contrast agent-relative concentration DCE (dynamic contrast enhanced)-MR imaging was used with a T1-weighted gradient-echo sequence. FLASH protocol was used with TE = 1.5 ms, TR = 12500 ms, flip angle 15°, 600 repetitions and identical matrix, resolution and FOV than T1 map. 0.025 mM/kg of gadoteridol was administered after the first 100 repetitions were acquired as baseline. Altogether, the MRI session lasted for 50 min approximately. After that, mice were returned to their home cage under close supervision until they were recovered from the anesthesia.

T1 maps were calculated in Paravision 6.0 software (Bruker BioSpin, Ettlingen, Germany). Later these maps were processed with custom-made algorithms programmed in Matlab (The MathWorks, Inc, Natick, MA, USA). A binary mask was manually drawn over the T1 map in order to segment the muscle in both legs. The first 40 volumes of the DCE acquistion were removed to assure the signal stabilization. Also, the last 50 volumes were discarded to avoid second pass effects. The slices of the temporal acquisition were spatially smoothed with a Gaussian filter (standard deviation = 0.5) and temporal smoothed with a moving average of 25 neighbors. The baseline of the signal was considered using the 20 volumes after the 10th. The signal intensities of the temporal acquisitions were converted to gadolinium concentrations using the method described in^63–65^. The T1 map acquired before the DCE was used as reference values for the magnetization and the Gadoteridol relaxivity was considered to be 3.35 s^−1^mM^−1 ^^65^. Finally, the obtained concentration curves were also smoothed with a moving average of nine neighbors. From the concentration curves, different parameters were estimated, such as the time to peak (TTP), the bolus arrival time (BAT) (using the method described in Cheong et al.^66^, relative time to peak (rTTP) (considering the BAT as starting point), the wash in and wash out slopes, and the area under the gadolinium concentration curve (AUC).

### Mouse-induced tumor model

LLC1 (Mouse Lewis lung cancer cells) (ATCC, Manassas, VA, USA) were cultured in Dulbecco’s Modified Eagle Medium (DMEM) supplemented with 10% fetal calf serum, 50 U/ml penicillin and 50 µg/ml streptomycin in humified atmosphere at 37 °C and 5% CO_2_. Syngeneic LLC1 tumor cells (1 × 10^5^) were subcutaneously injected into the flank of DHX15^+/−^ and wild-type mice. Primary tumor growth was controlled during the first 3 weeks. Primary tumors were surgically removed 21 days after seeding. Tumor volume was calculated by following formula: V = 4/3 × π × [length × depth × width]. Primary tumors were fixed in 4% PFA and cryoconserved in tissue-tek O.C.T. compound (Sakura, Flemingweg, Netherlands). The Post-surgical metastasis model was performed as follows: Two weeks after primary tumor removal, LLC1 injected mice showed distant metastasis formed in the lungs. Tile scan images of haematoxylin-eosin (H&E) stained paraffin lung sections were visualized using a microscope system (Nikon Eclipse E600, Kanagawa, Japan) and the percentage of the pulmonary metastatic area as percent of total lung area was measured with Image J software (ImageJ version 1.52b; National Institutes of Health, Bethesda, MD, USA).

### DHX15 silencing in liver endothelial cells and hepatocytes

The silencing of *DHX15* was carried out in mouse primary hepatic endothelial cells and mouse primary hepatocytes both immortalized with the SV40 virus (LEC and Hep; abmGood, Richmond, Canada), through shRNA by lentiviral infection (Dharmacon, Lafayette, Colorado, USA). The SMARTvector incorporated the bipartite 3G Tet-On® induction system, an inducible system with minimal basal expression and potent activation after induction with doxycycline. Endothelial cells were cultured in Microvascular Endothelial Cell Growth Complete Medium and hepatocytes in Complete Hepatocyte Medium (Pelobiotech, Planegg, Germany) in humified atmosphere at 37 °C and 5% CO_2_.

### Proteome and transcriptome analysis of siL-DHX15-LECs

For proteomics, proteins from non-silenced and DHX15-silenced LECs (1 mg/mL) were extracted in 100 mM NH_4_HCO_3_, 8 M urea, 2.5 mM sodium pyrophosphate, 1 mM sodium orthovanadate and 1 mM β-glycerol phosphate buffer. Samples were reduced with dithiothreitol (37 °C, 60 min) and alkylated in the dark with iodoacetamide (25 °C, 30 min). The resulting protein extract was first diluted to 2 M urea with 200 mM ammonium bicarbonate for digestion with endoproteinase LysC (1:10 w:w, 37 °C, o/n, Wako, cat # 129-02541), and then diluted 2-fold with 200 mM ammonium bicarbonate for trypsin digestion (1:10 w-w, 37 °C, 8 h, Promega cat # V5113). After digestion, peptide mix was acidified with formic acid and desalted with a MicroSpin C18 column (The Nest Group, Inc) prior to LC-MS/MS analysis. Samples were analyzed using a LTQ-Orbitrap Fusion Lumos mass spectrometer (Thermo Fisher Scientific, San Jose, CA, USA) coupled to an EASY-nLC 1000 (Thermo Fisher Scientific (Proxeon), Odense, Denmark). Peptides were loaded directly onto the analytical column and were separated reversed-phase chromatography with a 90-min gradient (0–35% ACN) in a 50-cm column with an inner diameter of 75 μm, packed with 2 μm C18 particles spectrometer (Thermo Scientific, San Jose, CA, USA). Acquired spectra were analyzed with ProteomeDiscoverer software (v2.0, Swiss-Prot mouse database as in November 2016, 16831 entries). Protein abundances were estimated with the average of the area corresponding to the three most intense peptides. Protein abundance estimates were then log-transformed, normalized by the median, and a fold change, and an adjusted *p-*value was calculated with Perseus 1.5.6.0.

The transcriptome analysis was carried out using RNAseq (HiSeq, Illumina). Total RNA from *Mus musculus* was quantified by Qubit® RNA BR Assay kit (Thermo Fisher Scientific) and the RNA integrity was estimated by using RNA 6000 Nano Bioanalyzer 2100 Assay (Agilent). The RNASeq libraries were prepared with KAPA Stranded mRNA-Seq Illumina® Platforms Kit (Roche-Kapa Biosystems) following the manufacturer´s recommendations. Briefly, 500 ng of total RNA was used as the input material, the poly-A fraction was enriched with oligo-dT magnetic beads and the mRNA was fragmented. The strand specificity was achieved during the second strand synthesis performed in the presence of dUTP instead of dTTP. The blunt-ended double-stranded cDNA was 3´-adenylated and Illumina indexed adapters (Illumina) were ligated. The ligation product was enriched with 15 PCR cycles and the final library was validated on an Agilent 2100 Bioanalyzer with the DNA 7500 assay. The libraries were sequenced on HiSeq 4000 (Illumina, Inc) in paired-end mode with a read length of 2 × 76 bp using HiSeq 4000 SBS kit in a fraction of a HiSeq 4000 PE Cluster kit sequencing flow cell lane, following the manufacturer’s protocol. Image analysis, base calling, and quality scoring of the run were processed using the manufacturer’s software Real-Time Analysis (RTA 2.7.6) and followed by the generation of FASTQ sequence files by CASAVA. RNA-seq reads were mapped against the mouse reference genome (GRCm38) using STAR version 2.5.2a^67^ with ENCODE parameters for long RNA. Genes were quantified with RSEM version 1.2.28^68^ using the gencode M12 version. Differential expression analysis was performed with DESeq2 version 1.18^69^ with default parameters. Differential alternative splicing was performed with rMATS^70^. Significant splicing events with FDR < 5%, absolute inclusion difference >5% and >70 number of reads were considered significant.

Signaling pathways altered by DHX15 deficiency were modeled using Ingenuity Pathway Software (Ingenuity®Systems, Inc., Redwood City, USA). The resulting *p-values* obtained by the Ingenuity Pathways Knowledge Base were adjusted for multiple comparisons using Benjamini and Hochberg’s method.

### Separation of respiratory complexes and supercomplexes by Clear-Native Page (CN-PAGE) and in-gel activity of Complex I

Solubilization of mitochondrial membranes by detergents, CN-PAGE, and staining was performed as described by Jha et al.^71^ with minor modifications. For this, after the mitochondria isolation from wild-type and siL-DHX15-LEC, 150 µg of mitochondrial protein were suspended in a low-salt buffer (50 mM NaCl, 50 mM imidazole, pH 7.0) and solubilized with digitonin (8 g/g protein, for solubilization of respiratory chain supercomplexes). Immediately after the electrophoretic run (4–13% gradient polyacrylamide gels), enzymatic colorimetric reaction was performed. Complex I activity was determined by incubating the gel with 2 mM Tris-HCl pH = 7.4, 0.1 mg/mL NADH, and 2.5 mg/mL nitro blue tetrazolium (NTB) at room temperature. The original color of the complex I was preserved by fixing the gels in 50% methanol and 10% acetic acid. After gel scanning, the intensity of each band was quantified by Image J software (ImageJ version 1.52b; National Institutes of Health, Bethesda, MD, USA).

### Mouse trachea whole-mount immunofluorescence staining

Trachea tissue was fixed overnight in a solution containing 80% methanol and 20% DMSO at −20 °C, and permeabilized in Tris-buffered saline (TBS) containing 2% BSA and 0.1% Triton for 5 h. Tissue was incubated with rat anti-mouse CD31 antibody (BD Pharmigen, Franklin Lakes, New Jersey, USA) at a 1:100 dilution overnight, washed 4 times with TBS and blocked again with TBS containing 2% BSA and 0.1% Triton for 3 h. Finally, the tissue was incubated with Alexa Fluor 555-conjugated rat anti-mouse antibody (Thermo Fisher, Waltham, MA, USA) in a 1:500 dilution overnight and washed eight times with TBS. The immunofluorescence signal was visualized using an immunofluorescence microscope system (Nikon Eclipse E600, Kanagawa, Japan).

### Mouse fluorescence lymphangiography

To evaluate the lymphatic drainage in peripheral areas, 0.05 mL of FITC-Dextran 2000 KDa (Sigma-Aldrich, Darmstadt, Germany) was subcutaneously injected in ear, tail, and footpad of wild-type and transgenic mice, with a 30-gauge needle. Immediately after injection, fluorescence images of subcutaneous lymphatic drainage were visualized on a fluorescence stereomicroscope system (Leica Microsystems, Heerbrugg, Switzerland).

### Western blot experiments

Cell lysates were prepared in a lysis buffer (Tris–HCl 20 mM pH = 7.4 containing 1% Triton X-100, 0.1% SDS, 50 mM NaCl, 2.5 mM EDTA, 1 mM Na_4_P_2_O_7_·10H_2_O, 20 mM NaF, 1 mM Na_3_VO_4_, 2 mM Pefabloc and Complete^®^ from Roche). Proteins were separated on a 10% SDS-polyacrylamide gel (Mini Protean III; Biorad, Hercules, CA, USA) and transferred for 2 h at 4 °C to nitrocellulose membranes (Transblot Transfer Medium; Biorad, Hercules, CA, USA) that were stained with Ponceau-S red as a control for protein loading. Then, the membranes were incubated at 4 °C with the following antibodies: mouse monoclonal anti-mouse DHX15 (Santa Cruz Biotechnology, Dallas, Texas, USA), rabbit polyclonal anti-mouse CD31, rabbit anti-mouse β-actin HRP Conjugate (Cell Signaling, Danvers, MA, USA), mouse monoclonal anti-mouse eNOS (BD Bioscience, San Jose, CA, USA) or rabbit monoclonal anti-mouse Ndufs1 (Abcam, Cambridge, UK) overnight in a 1:1000 dilution. Next, the membranes were incubated with goat anti-rabbit peroxidase-conjugated secondary antibody or goat anti-mouse peroxidase-conjugated secondary antibody at a 1:2000 dilution (Cell Signaling, Danvers, MA, USA) for 1 h at room temperature. The bands were visualized by chemiluminescence (Clarity Western ECLS substrate; Biorad, Hercules, CA, USA). The intensity of each band was quantified by Image J software (ImageJ version 1.52b; National Institutes of Health, Bethesda, MD, USA). Band intensities were measured and normalized to the indicated sample (shown as 1.00) on the same membrane. The molecular mass of the proteins was determined by comparing their electrophoretic mobility with that of the proteins contained in the Precision Plus Protein Standards Dual Color market (Biorad, Hercules, CA, USA).

### Immunocytofluorescence and uptake of oxiLDL

For *immunocytofluorescence*, LECs were fixed with 4% paraformaldehyde and blocked with 5% normal goat serum. Next, cells were incubated with rabbit anti-mouse podoplanin (Sigma Chemical, St Louis, Missouri, USA) for 1 h in a 1:200 dilution. The respective secondary antibody was Alexa Fluor 488-conjugated goat anti-mouse (Molecular Probes, Invitrogen, San Diego, California, USA). In addition, 4′,6-diamidino-2-phenylindole (DAPI, Vectashield, Vector laboratories, Burlingame, Ca) was used to counterstain cell nuclei. Immunofluorescence was visualized with an immunofluorescence microscope system (Nikon Eclipse E600, Kawasaki, Kanagawa, Japan).

For the *uptake of oxiLDL*, cells were incubated with Alexa Fluor 488-conjugated AcLDL (Thermo Fisher, Waltham, MA, USA) at a concentration of 10 µg/mL for 3 h. Then, cells were washed three times with PBS and fixed with 4% paraformaldehyde. Fluorescence was visualized with an immunofluorescence microscope system (Nikon Eclipse E600, Kawasaki, Kanagawa, Japan).

### Real-time quantitative PCR (RT-qPCR)

Total RNA was extracted from cultured LECs using the Trizol reagent (Life Technologies, Carlsbad, CA, USA). One microgram of total RNA was reverse transcribed using First Strand cDNA Synthesis Kit (Roche, Mannheim, Germany). Subsequently, complementary DNA samples were amplified for 30–35 cycles (94 for 30 s, 55–60 °C for 30 s, and 72 °C during 1 min; LigthCycler 480-Roche Diagnostics). To normalize the results, *HPRT* gene was used as reference. Specific primers for amplification of the complementary DNA can be found in Supplementary Table 1.

### Cell proliferation assay

The bromodeoxyuridine (BrdU) cell proliferation assay kit (BrdU Flow kit; BD Pharmigen, Franklin Lakes, New Jersey, USA) was used to measure the incorporation of BrdU during DNA synthesis following the manufacturer’s protocols. Briefly, when cells reached about 60–70% of confluence, BrdU (10 μM) was added to the culture medium for 1 h. Then, the BrdU-labeled cells were fixed and the DNA was denatured in fixative solution for 1 h at 37 °C. Next, the cells were incubated with Alexa Fluor 555-conjugated anti-BrdU antibody for 1 h at room temperature. Immunofluorescence was detected by flow cytometry (LSRFortessa; BD Bioscience, San Jose, CA, USA).

### Wound healing assay

The wound-healing assay was performed in wild-type and siL-DHX15-LEC, and in primary lymphatic endothelial cell isolated from wild-type and DHX15^+/−^ mice. Cells were cultured in six-well plates and incubated at 37 °C until they reached confluency. In the case of wild-type and siL-DHX15-LEC, when they reached around 75% confluency were treated or not with ATP (5 µM) or Pyruvate (2 mM) (Sigma-Aldrich, Darmstadt, Germany). Next day, when cells were confluent, the straight scratch wound was performed with a 100 μL sterile pipette tip. The scratched areas were photographed using an inverted phase-contrast microscope (Olympus IX51, Tokyo, Japan) at 0, 7, and 24 h, respectively.

### Quantification of intracellular ATP

The cellular production of ATP was determined in non-silenced and DHX15-silenced endothelial cells, and in primary mouse liver sinusoidal endothelial cells isolated from wild-type and DHX5^+/−^ mice with a luminescence assay (Cayman Chem, Ann Arbor, MI, USA) according to the manufacturer’s instructions.

### Tumor tissue immunofluorescence

Tissues were fixed in 4% paraformaldehyde, cryoprotected overnight in a 30% sucrose solution and embedded in optimal cutting temperature medium. Next, 2-µm frozen sections were rehydrated, blocked with 5% normal goat serum and incubated with rat anti-endomucin or rabbit anti-Lyve-1 (Abcam, Cambridge, UK) as a primary antibody overnight at 4 °C. The binding sites of the primary antibodies were revealed with Alexa Fluor 488-conjugated goat anti-rat or Alexa Fluor 488-conjugated goat anti-rabbit (Thermo Fisher, Waltham, MA, USA). Tissues for which immunostaining was performed without primary antibodies were used as negative controls. Slides were mounted using Vectashield (Vector Laboratories, Burlingame, CA, USA) and samples were visualized with a fluorescence microscope (Nikon Eclipse E600, Kanagawa, Japan).

### Isolation of primary mouse liver endothelial cells (MLiECs)

Freshly isolated primary MLiECs were obtained from the liver of the control and DHX15^+/−^ mice. MLiECs were purified after collagenase A retrograde perfusion (Roche Diagnostics, Basel, Switzerland) and Nycodenz gradient (Sigma-Aldrich, Darmstadt, Germany). The purification was optimized using magnetic beads CD146^+^ (MACS system, Miltenyi Biotec, Bergisch-Gladbach, Germany). Cells were cultured in Microvascular Endothelial Cell Growth Complete Medium (Pelobiotech, Planegg, Germany) in humified atmosphere at 37 °C and 5% CO_2_.

### Isolation of primary lymphatic endothelial cells (LyECs)

Freshly isolated primary LyECs were obtained from the mesentery of the control and DHX15^+/−^ mice. LyECs were isolated after incubation of mesenteric lymphatic tissue mucosa in collagenase A (Roche Diagnostics, Basel, Switzerland) at 37 °C. The purification was optimized using rabbit anti-rat podoplanin antibody (Sigma Chemical, St. Louis, Missouri, USA) in a 1:100 dilution as the primary antibody and microbeads coupled with a secondary goat anti-rabbit antibody (MACS system, Miltenyi Biotec). The cells were grown in Microvascular Endothelial Cell Growth Complete Medium (Pelobiotech, Planegg, Germany) in humified atmosphere at 37 °C and 5% CO_2_.

### Visualization of mouse embryo yolk sacs

Embryos with the yolk sac were harvested at E10.5 and put in 4% paraformaldehyde solution at 4 °C. Images were acquired immediately using a visible stereomicroscope (Leica Microsystems, Heerbrugg, Switzerland).

### Complex I enzyme activity

The complex I enzyme activity was determined in non-silenced and DHX15-silenced endothelial cells by a colorimetric enzymatic reaction assay (Abcam, Cambridge, UK), a according to the manufacturer’s instructions.

### Akt activity assay

Akt activity was determined in non-silenced and DHX15-silenced endothelial cells using a commercial kinase activity assay (Abcam, Cambridge, UK), according to the manufacturer’s instructions. In brief, Akt was immunoprecipitated using Akt-specific antibody and protein A beads. Then, the Akt activity was determined in a kinase reaction using recombinant GSK-3 as substrate. Finally, the levels of phosphorylation of the GSK-3 were analyzed by western blot using an anti-phospho-GSK-3 specific antibody.

### OCR in endothelial cells and hepatocytes

In vivo real-time mitochondrial respiration (OCR) was monitored with the Seahorse XFe24 Flux Analyser (Seahorse Bioscience) according to the manufacturer’s instructions. Non-silenced and DHX15-silenced endothelial cells were seeded at 15,000, 20,000 and 30,000 cells per well in 3–4 replicates for each density in a 24-well plate custom designed for XFe24 analysis and cultured overnight in endothelial culture media. For assessment of the real-time OCR, cells were incubated with unbuffered assay media (XF Media Base with 10 mM glucose, 2mM L-glutamine and 2 mM sodium pyruvate) followed by sequential injection of oligomycin (final concentration 2 µM), carbonyl cyanide-4-(trifluoromethoxy) phenylhydrazone (FCCP; final concentration 1 µM), and rotenone plus antimycin A (final concentration 1 µM each).

Non-silenced and DHX15-silenced hepatocytes were seeded at 50,000 cells per well in 3–4 replicates for each density in a 24-well plate custom designed for XFe24 analysis and cultured overnight in endothelial culture media. For assessment of the real-time OCR, cells were incubated with unbuffered assay media (XF Media Base with 5.5 mM glucose, 2mM L-glutamine and 1 mM sodium pyruvate) followed by sequential injection of oligomycin (final concentration 1 µM), carbonyl cyanide-4-(trifluoromethoxy) phenylhydrazone (FCCP; final concentration 0.5 µM), and rotenone plus antimycin A (final concentration 1 µM each). The results from the mitochondrial respiration assay were analyzed by the XFe wave software (Seahorse Bioscience Inc., MA), and displayed as OCR normalized to μg of total protein following BCA protein assay (pmol/min per µg of protein).

### Survival analysis

Survivals were plotted on Kaplan–Meier curves and compared using the log-rank test. Total survival at 5 weeks after LLC1 cell implantation was the chosen end point.

### Unsupervised hierarchical clustering analysis

RNAseq results were filtered and standardized before undergoing unsupervised hierarchical clustering analysis in Cluster^72^. Results were visualized with the Java Treeview software^73^.

### Gene set enrichment-gene ontology analysis (GSEA-GO)

GSEA-GO analysis includes three categories: molecular function, biological process, and cellular component. GO analysis was performed through gseGO function in clusterProfiler package^74^. The adjusted *p*-value < 0.05 was set as the cut-off criteria. Selected GO terms were visualized using ridgeplot representation.

### Statistics and reproducibility

In the case of homoscedasticity and normally distributed data (assessed by Shapiro–Wilk test), groups were compared using a two-sided Student *t* test or analysis of variance for independent samples. For other types of data, Mann–Whitney *U* test, or Kruskal–Wallis test was used. Tukey’s test (with analysis of variance) or Dunn’s test (with Kruskal–Wallis) was used as a post hoc test to perform pairwise comparisons. The statistical analysis of contingency tables for proportions was performed using the Fisher’s exact test. Differences were considered to be significant at a *p*-value < 0.05. The data are presented as the mean ± standard error of the mean. No sample-size calculation was performed. However, all the single conclusions stated in our study were supported by statistical differences obtained from the results of at least two different experimental methodologies designed to demonstrate differences for the same biological process.

All the statistical analyses were performed using the GraphPad software v 5.0 or public libraries from the Comprehensive R Archive Network (CRAN; http://CRAN.R-project.org) rooted in the open-source statistical computing environment R, version 3.6 (http://www.R-project.org/).

### Reporting summary

Further information on research design is available in the Nature Research Reporting Summary linked to this article.

## Supplementary information

Supplementary Information (new)

Description of Additional Supplementary Files

Supplementary Data 1

Supplementary Data 2

Supplementary Data 3

Supplementary Data 4

Supplementary Data 5

Reporting Summary

## Data Availability

All relevant data are deposited into a public repository and accession codes are provided in the published article. Full information regarding the antibodies used can be found in Supplementary Table 2. All source data corresponding to all graphs and plots from Figs. 1–7 can be found in Supplementary Data 5. Uncropped Western Blots can be found in Supplementary Figs. 11, 12, and 13. The raw proteomics data have been deposited to the PRIDE repository with the dataset identifier PXD018104. The raw data set of RNAseq results is available at the data repository Gene Expression Omnibus using the accession display GSE183263. All other data are available from the corresponding author on reasonable request. All unique materials (DHX15 deficient mice, and transfected cell lines) are readily available from the authors. *DHX15* gene-deficient zebrafish is readily available from standard commercial sources (ZeClinics).
