# Supplementary material for: The loss of DHX15 impairs endothelial energy metabolism, lymphatic drainage and tumor metastasis in mice
Source: Commun Biol. 2021 Oct 15;4:1192. doi: 10.1038/s42003-021-02722-w (PMC8519955; doi:10.1038/s42003-021-02722-w)
Supplement: Supplementary file 1 — Supplementary Information (new) [file 42003_2021_2722_MOESM1_ESM.pdf]

SUPPLEMENTARY INFORMATION

The loss of *DHX15* impairs endothelial energy metabolism, lymphatic drainage and tumor metastasis in mice

Jordi Ribera[1], Irene Portolés[1], Bernat Córdoba-Jover[1], Juan Rodríguez-Vita[1,2], Gregori Casals[1], Bernardino González-de la Presa[1], Mariona Graupera[3], Estel Solsona-Vilarrasa[4], Carmen Garcia-Ruiz[4,5], José C. Fernández-Checa[4,5], Guadalupe Soria[6], Raúl Tudela[6], Anna Esteve-Codina[7], Guadalupe Espadas[8], Eduard Sabidó[8], Wladimiro Jiménez[1,9], William Sessa[10], Manuel Morales-Ruiz[1,9,*]

[1]Biochemistry and Molecular Genetics Department, Hospital Clínic of Barcelona, Institut d'Investigacions Biomèdiques August Pi i Sunyer (IDIBAPS), Centro de Investigación Biomédica en Red de Enfermedades Hepáticas y Digestivas (CIBERehd), Barcelona, Spain.

[2]German Cancer Research Center, Heidelberg, Germany.

[3]Vascular Signalling Laboratory, Program Against Cancer Therapeutic Resistance (ProCURE), Institut d'Investigació Biomèdica de Bellvitge (IDIBELL). CIBERonc, Barcelona, Spain.

[4]Cell Death and Proliferation, Institute of Biomedical Research of Barcelona (IIBB), Consejo Superior Investigaciones Científicas (CSIC), Liver Unit, Hospital Clínic, IDIBAPS, Universitat de Barcelona, Barcelona, 08036 Spain; CIBERehd, Instituto de Salud Carlos III, Madrid, 28029, Spain.

[5]USC Research Center for ALPD, Keck School of Medicine, Los Angeles, United States, CA 90033.

[6]Experimental 7T-MRI Unit, IDIBAPS, Barcelona, Spain, CIBERbbn, University of Barcelona, Barcelona, Spain.

[7]CNAG-CRG, Centre for Genomic Regulation (CRG), Barcelona Institute of Science and Technology (BIST), Universitat Pompeu Fabra (UPF), Barcelona, Spain.

[8]Proteomics Unit, Centre for Genomic Regulation (CRG), The Barcelona Institute for Science and Technology, Universitat Pompeu Fabra, Barcelona, Spain.

[9]Department of Biomedicine-Biochemistry Unit, School of Medicine University of Barcelona, Barcelona, Spain.

[10]Department of Pharmacology, Department of Cardiology, Vascular Biology and Therapeutics Program, Yale University School of Medicine, New Haven, Connecticut, USA.

*** Corresponging author:** Dr. Manuel Morales-Ruiz, Department of Biochemistry and Molecular Genetics, Hospital Clinic of Barcelona, 170 Villarroel Street, 08036 Barcelona, Spain; e-mail: morales@clinic.cat

ORCID: Orcid.org/0000-0002-9074-2272

SUPPLEMENTARY FIGURES

Supplementary Figure 1. Characterization of the LEC cell line.

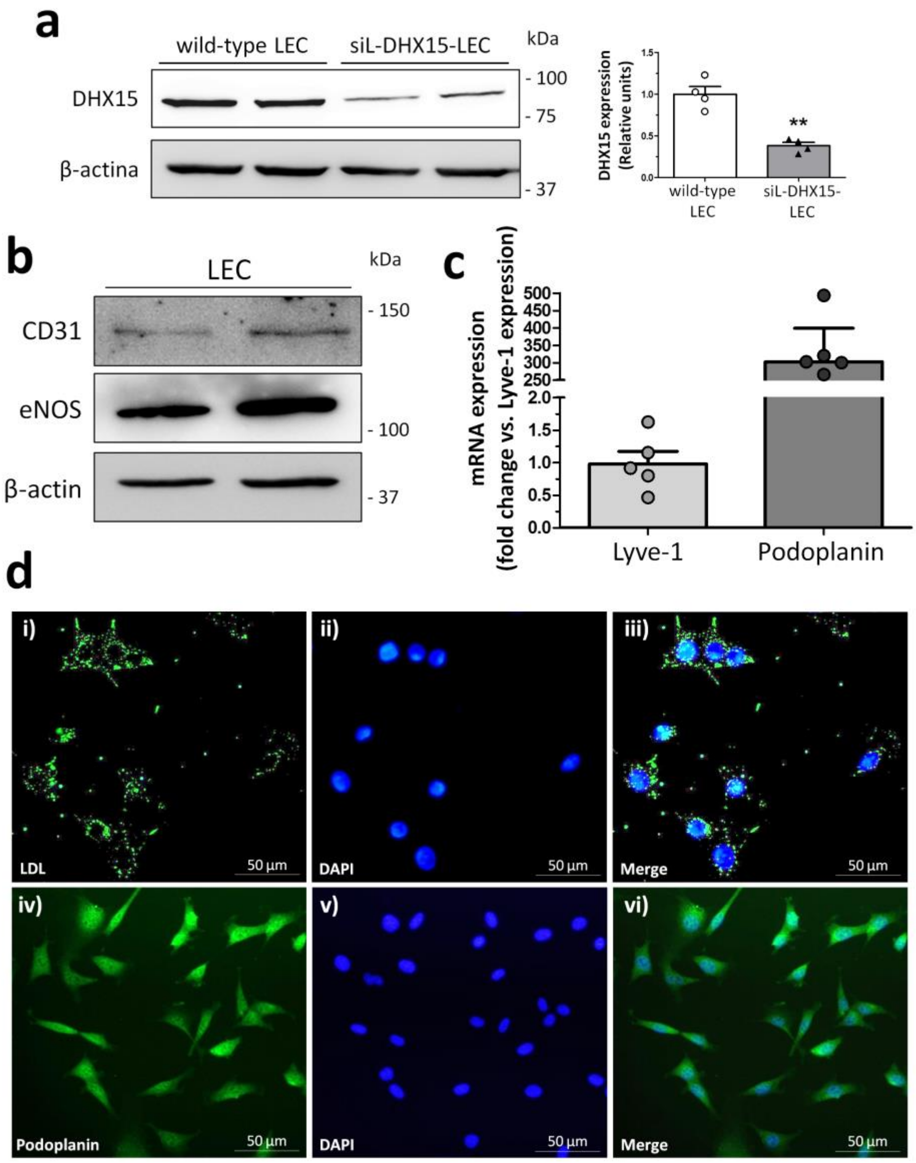

a) The expression of DHX15 protein was evaluated by western blot using cell lysates from wild-type and silenced DHX15 liver endothelial cells (siL-DHX15-LEC). β-actin was used as a loading control. The densitometric analysis of the protein expression is shown on the right bar graph. ***p*<0.01 *vs*. wild-type LEC, unpaired two-tailed Student's t-test (n=4 biologically independent samples for each condition). **b)** The expression of CD31 and eNOS protein was evaluated by western blot using cell lysates from liver endothelial cells (LEC). β-actin was used as a loading control. **c)** LEC were lysed in trizol and their mRNA expression was analyzed by RT-qPCR, as described in Methods. Graph show the expression levels for the genes *Lyve-1* and *Podoplanin*. mRNA levels are illustrated as fold change relative to *HPRT* mRNA levels (n=5 biologically independent samples). **d)** Oxidized low-density lipoprotein (oxLDL) uptake (upper panels i and iii; green) and immunostaining of the lymphatic endothelial marker podoplanin (lower panels iv and vi; green) are shown for LEC. Cell

nuclei were stained with DAPI (blue). Epifluorescence microscope, original magnification: 200X (n=3 independent experiments). All bar graphs are presented as mean ± SEM.

Supplementary Figure 2. Characterization of DHX15 heterozygous mouse.

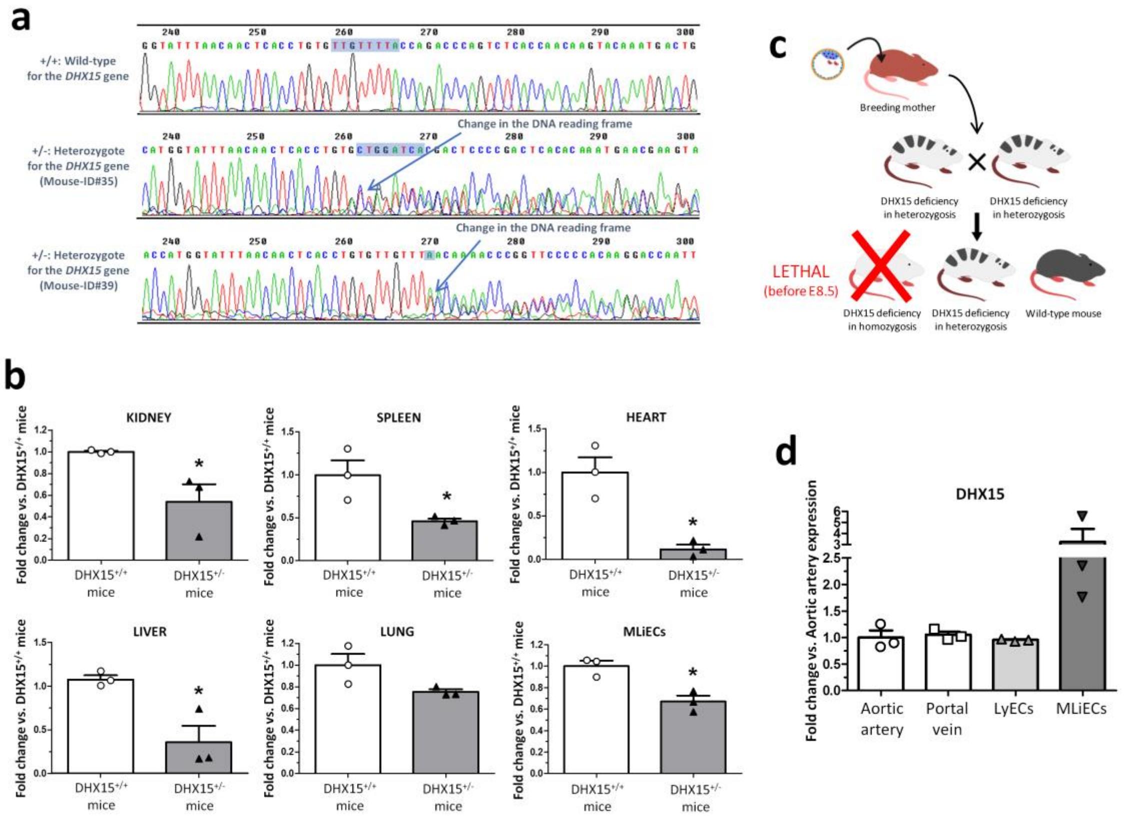

a) Comparison of sequencing chromatograms from wild-type and the heterozygote mouse of the *DHX15* gene obtained from the clones Mouse-ID#35 and Mouse-ID#39. The TALEN target site is highlighted within the box. The arrow shows the first base from which the DNA reading frame undergoes nonsense-mediated decay. **b)** Tissues or primary isolated cells from wild-type and DHX15$^{+/-}$ mice were lysed in Trizol® and their mRNA expression was analyzed by RT-qPCR, as described in Methods. The graph shows the expression level of the *DHX15* gene. mRNA levels are depicted as fold change relative to *HPRT* mRNA levels. *p<0.05 *vs*. Control, unpaired two-tailed Student’s t-test (n=3 biologically independent samples for each condition). **c)** Scheme showing the transgenic mouse generation from TALEN RNA injection in pronucleated oocytes. **d)** Aortic artery, portal vein, primary LyECs and primary MLiECs from wild-type mice mRNA expression was analyzed by real-time PCR. Graph shows the different expression levels for *DHX15* gene. mRNA levels are illustrated as fold change relative to *HPRT* mRNA levels (n=3 biologically independent samples for each condition). All bar graphs are presented as mean ± SEM.

Supplementary Figure 3. Characterization of isolated primary cells.

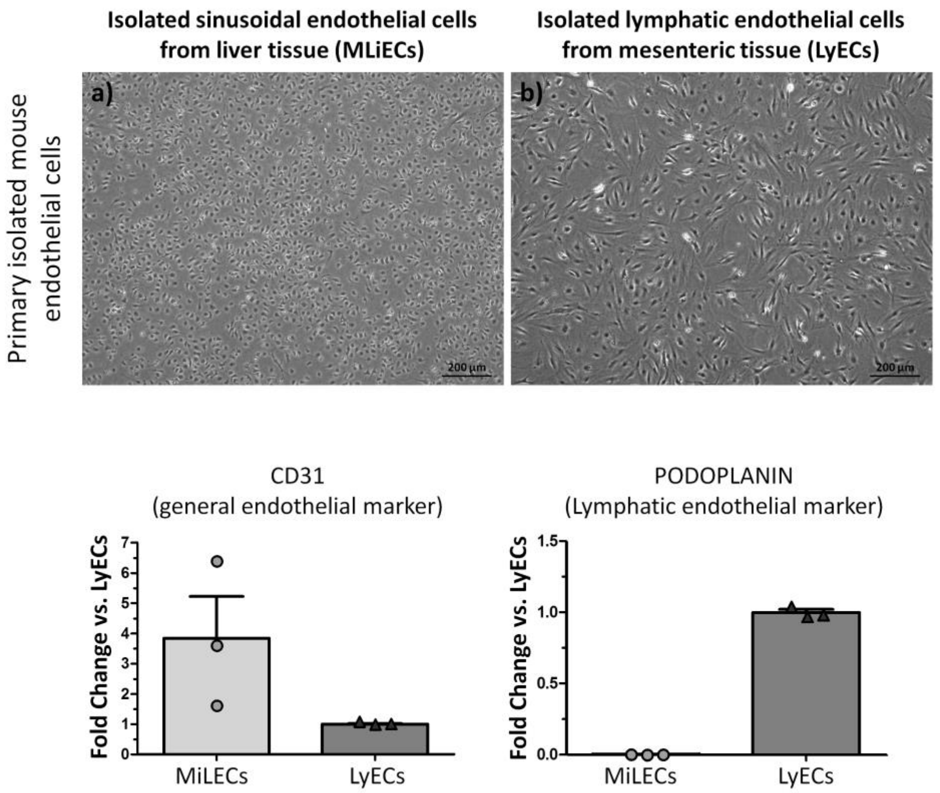

Representative bright fields for isolated mouse sinusoidal endothelial cells (panel a) and mouse lymphatic endothelial cells (panel b). Original magnification: x40. Bottom graphs show the different gene expression levels for endothelial markers (n=3 biologically independent samples for each condition). All bar graphs are presented as mean ± SEM.

Supplementary Figure 4. Colocalization of *DHX15* gene expression and vasculature in zebrafish embryos.

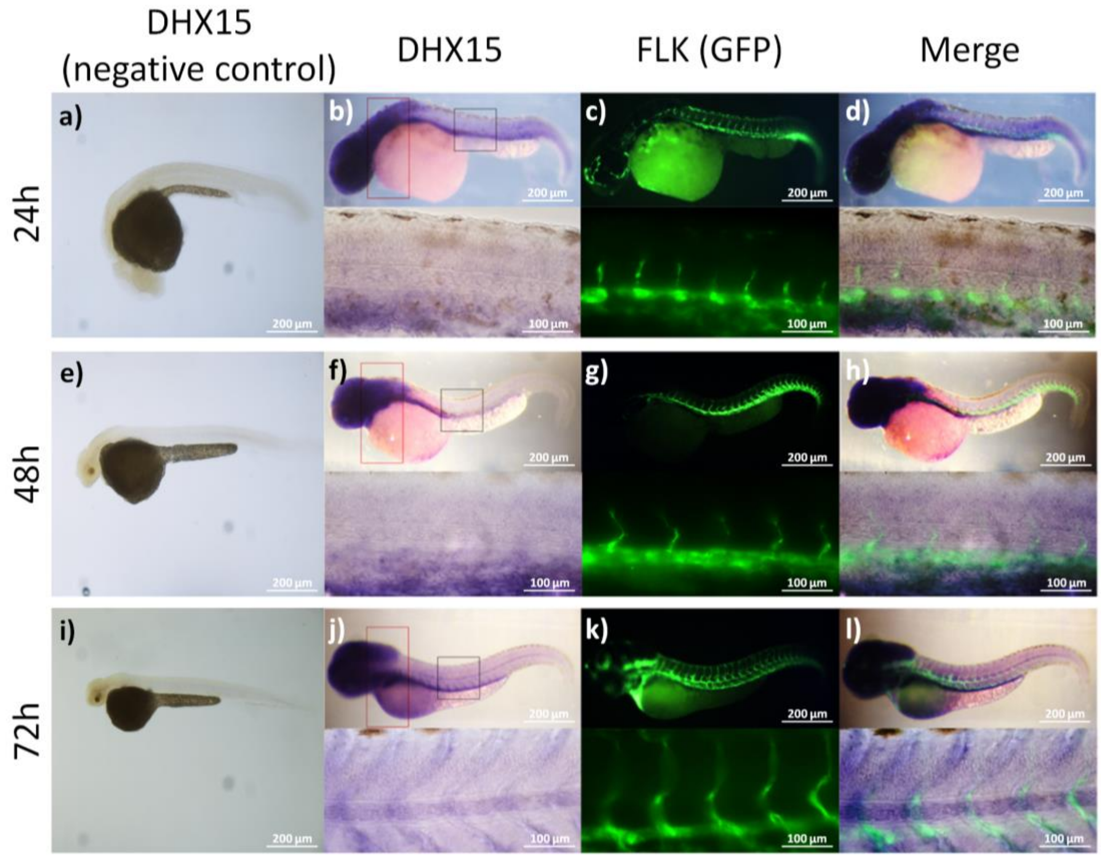

Representative results obtained from in situ hybridization using a labelled complementary RNA strand to localize the specific DHX15 sequence on whole-mount zebrafish embryos. Panels b, f and j show DHX15 mRNA localization (blue) in zebrafish embryos at 24, 48 and 72h of post-natal development, respectively. Panels c, g and k show the vascular anatomy (FLK1:EGFP; green) in zebrafish embryos at 24, 48 and 72h of post-natal development, respectively. Panels d, h and l show merged images of DHX15 mRNA and vasculature colocalization (green) in zebrafish embryos at 24, 48 and 72h of post-natal development, respectively. (n=15 zebrafish larvae). Negative controls of the in situ hybridization for DHX15 are shown in panels a, e, and i in zebrafish embryos at 24, 48 and 72h of post-natal development, respectively.

Supplementary Figure 5. Bioinformatics analysis of DHX15 knockdown in endothelial cells.

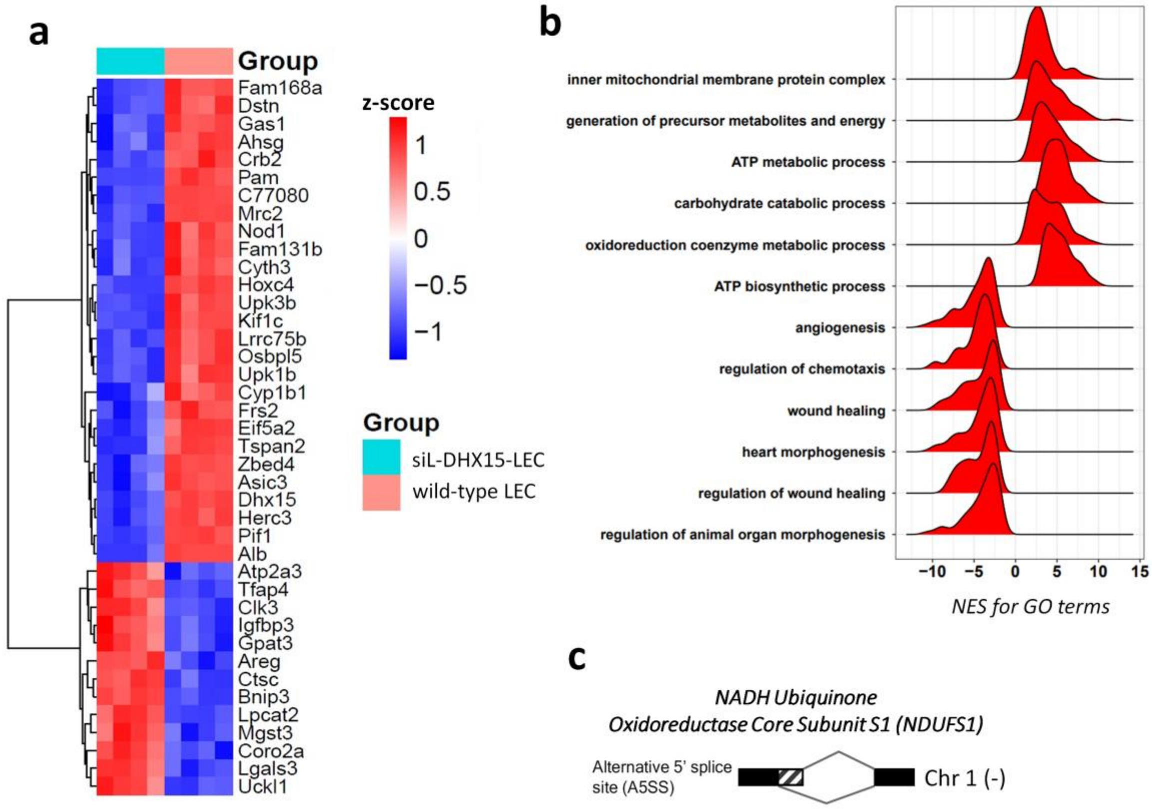

a) Hierarchical clustering analysis of differentially-regulated isoforms found in the wild-type and the DHX15-silenced LEC. The intensity of each color denotes the standardized ratio between each value and the average expression of each gene across all samples (z-score). Red pixels correspond to an increased abundance of mRNA in the indicated sample (n=4 biologically independent samples for each condition). Log2 Foldchange values are represented in the red-blue code bar shown on the right. **b)** Gene Set Enrichment based on Gene Ontology (GO) Analysis. Selected GO terms were visualized using ridge plot representation. Negative GO terms are underrepresented in siL-DHX15-LEC according to the normalized enrichment score (NES) for GO terms, while positive GO terms are overrepresented in siL-DHX15-LEC according to NES for GO terms. **c)** Alternative splicing was quantified as described in Methods. The diagram shows the significant splicing event occurring on the *NDUFS1* gene with an inclusion level of 6% and a FDR=0.02. The striped bar represents the lengthening of the alternative 5' limit size of exon 1 caused by the DHX15 silencing.

Supplementary Figure 6. Network analysis of molecular changes derived from DHX15 deficiency in LEC.

a

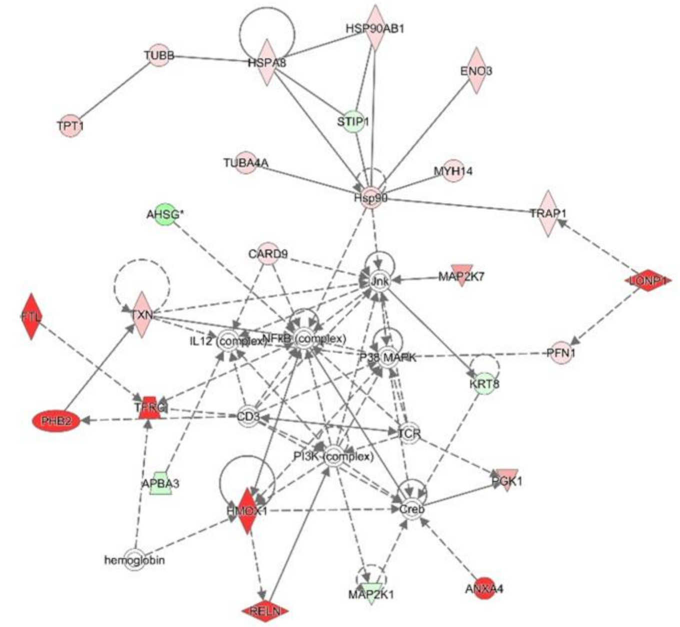

Network 1. Endocrine system disorders, organismal injury and abnormalities, and cancer; *Score 46*.

b

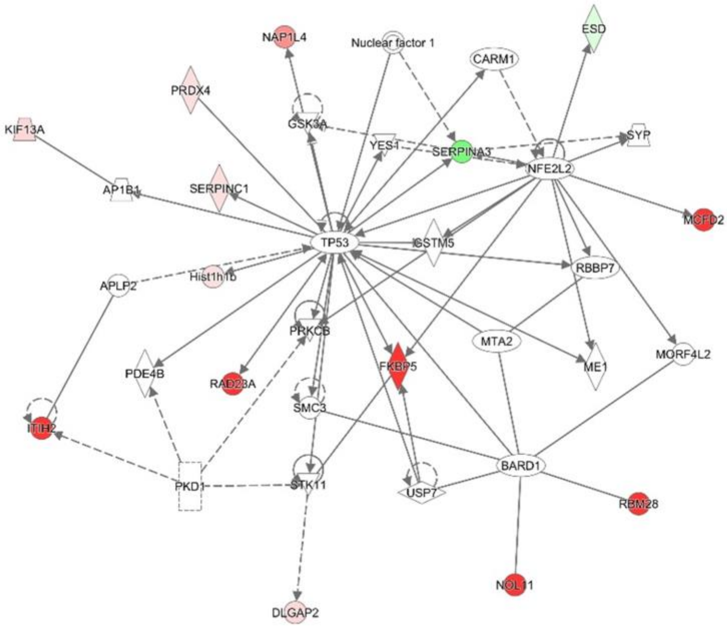

Network 2. Gastrointestinal disease, organismal injury and abnormalities, and carbohydrate metabolism; *Score 21.*

Networks of pathways obtained from the proteogenomic analysis of wild-type and silenced DHX15 liver endothelial cells (siL-DHX15-LEC) were algorithmically generated based on their connectivity. **a)** Network 1 is associated with the functional category of endocrine system disorders, organismal injury and abnormalities, and cancer. **b)** Network 2 is associated with the functional category of gastrointestinal disease, organismal injury and abnormalities, and carbohydrate metabolism. In both networks, the proteins are represented as nodes, and the biological relationship between two nodes is represented as an edge (line). A colored node indicates a protein that was detected by the proteogenomic screening (red: overexpressed and green: reduced expression in silenced-LEC). Nodes are displayed using various shapes that represent the functional class of the proteins. Edges with dashed lines show indirect interaction.

Supplementary Figure 7. Glycolytic pathway validation of RNA-seq results.

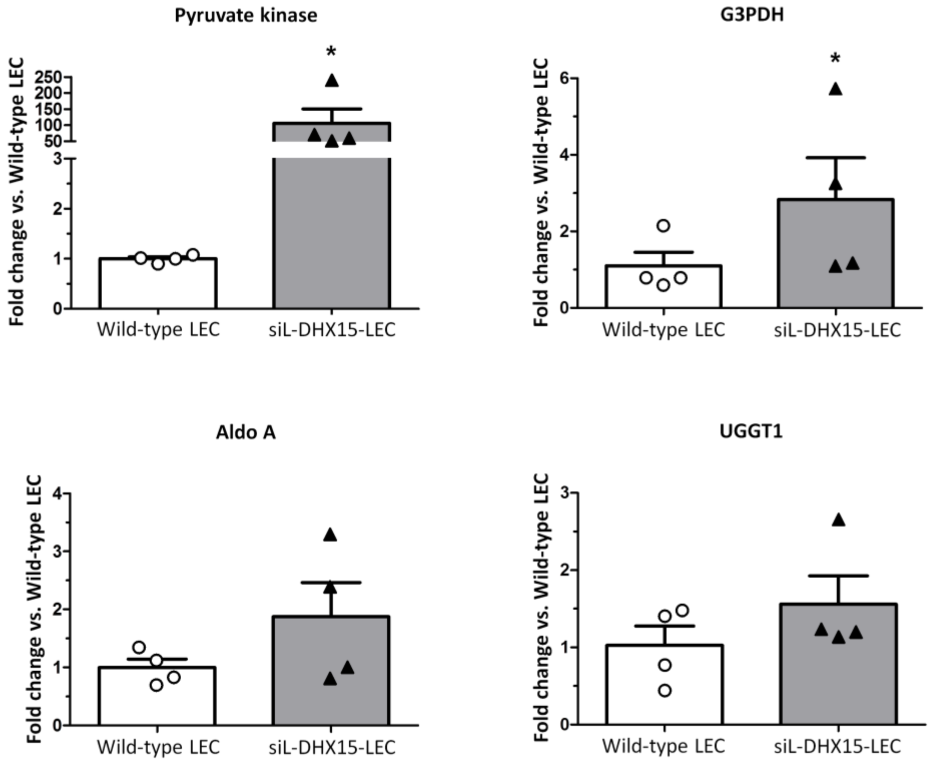

Cell lysates from wild-type and siL-DHX15-LEC were lysed in trizol and their mRNA expression was analyzed by real-time PCR, as described in Methods. Graph show the different expression levels for the corresponding genes: *Pyruvate kinase*, *G3PDH*, *Aldo A* and *UGGT1*. mRNA levels are illustrated as fold change relative to *HPRT* mRNA levels. **p*<0.05 *vs*. control (n=4 biologically independent samples for each condition). All statistical analyses were performed using unpaired two-tailed Student’s t-test. All bar graphs are presented as mean ± SEM.

Supplementary Figure 8. Mitochondrial oxygen consumption rate in DHX15-silenced hepatocytes.

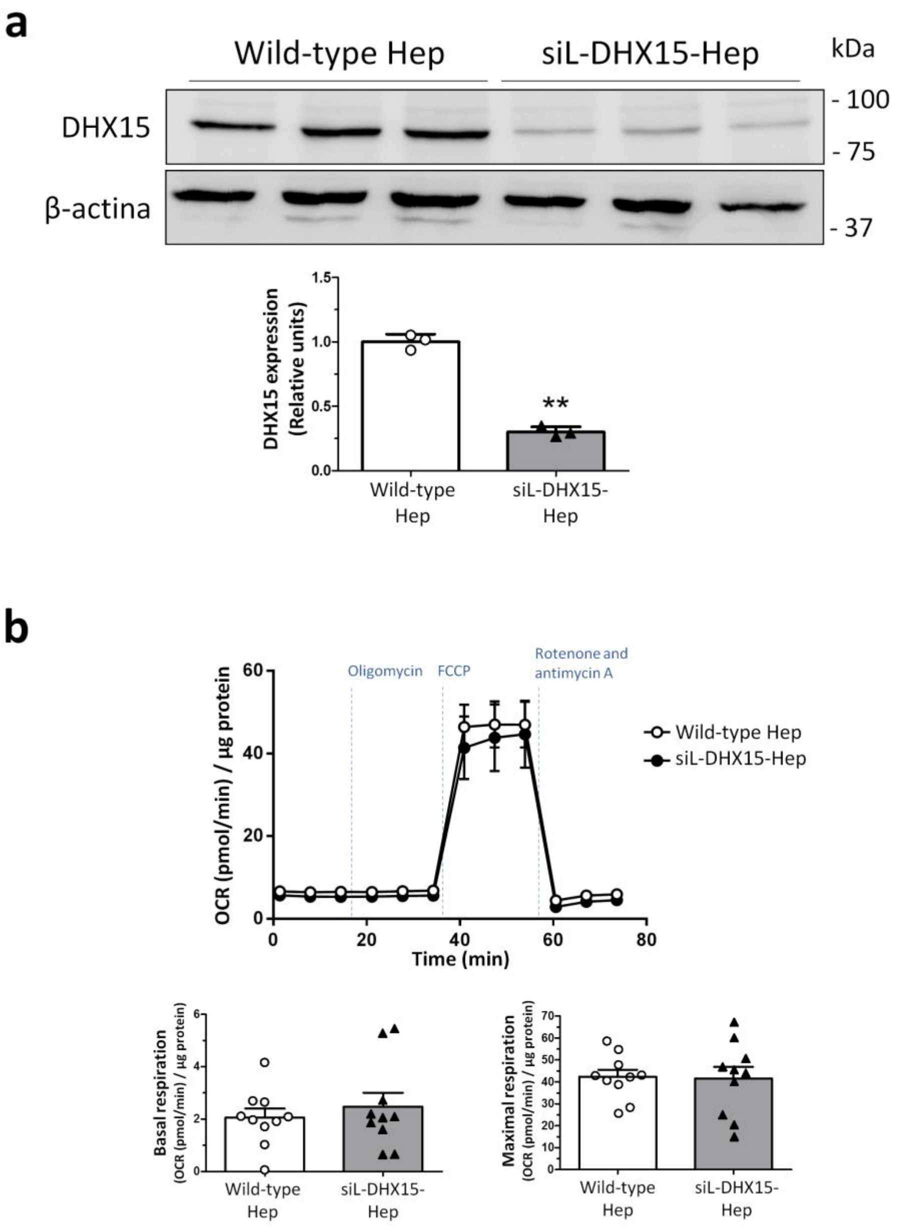

a) The expression of DHX15 protein was evaluated by western blot using cell lysates from wild-type and silenced DHX15 hepatocytes (siL-DHX15-Hep). β-actin was used as a loading control. The densitometric analysis of the protein expression is shown on the bottom bar graph. ***p*<0.01 *vs*. wild-type Hep (n=3 biologically independent samples for each condition). **b)** Wild-type and siL-DHX15-Hep were evaluated for basal, and maximal respiration in a mito-stress assay using a Seahorse XFe24 analyzer (n=10 independent Seahorse wells for each condition). OCR displayed is normalized to µg of total protein. All statistical analyses were performed using unpaired two-tailed Student's t-test. All bar graphs are presented as mean ± SEM.

Supplementary Figure 9. Functional characterization of DHX15 knockdown in endothelial cells.

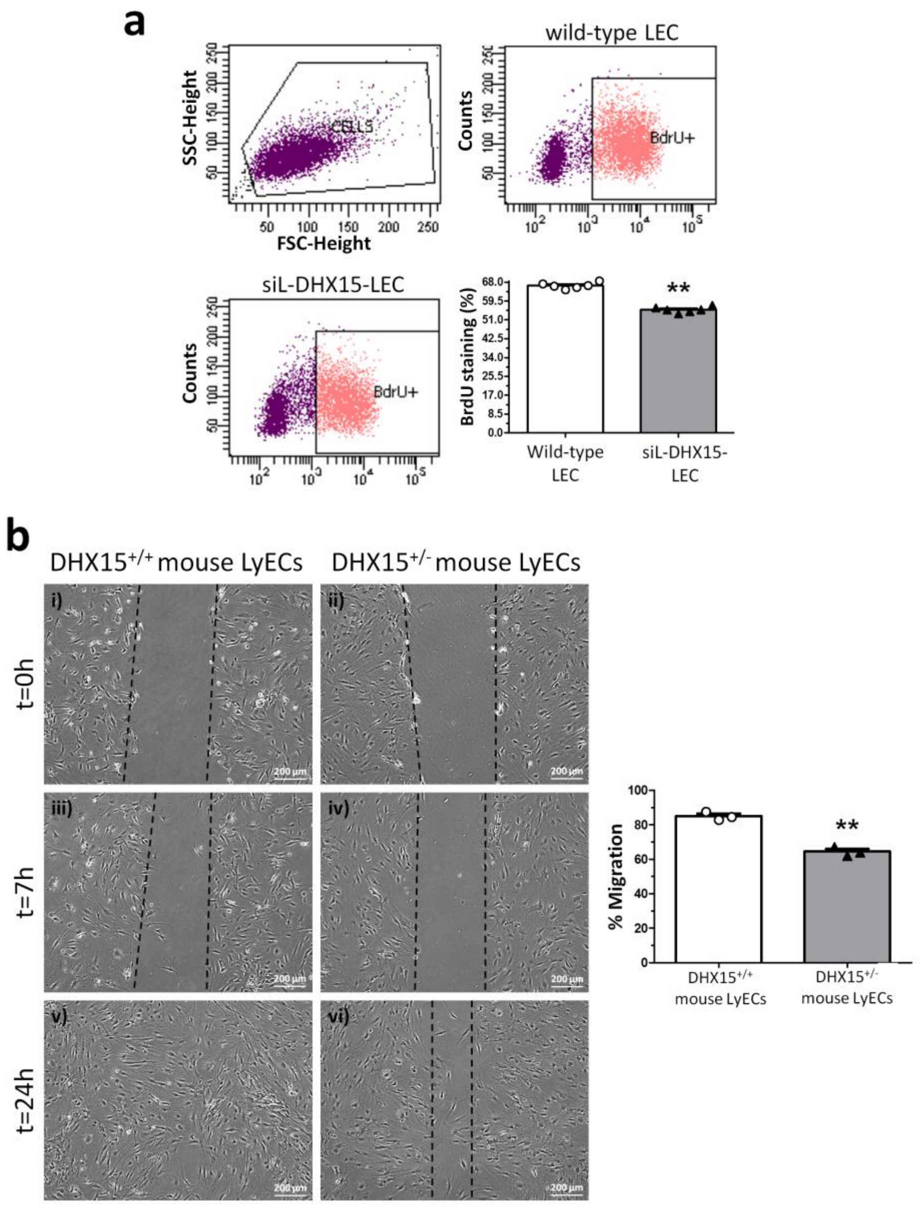

a) Representative figures of the proliferation assay performed in wild-type and silenced DHX15 liver endothelial cells (siL-DHX15-LEC). Bromodeoxyuridine (BrdU) incorporation was quantified by flow cytometry. First panel shows a dot-blot graph of the cell population. Cells within the oval scatter gate were analyzed. The negative control population was chosen from cells cultured in the absence of BrdU. The percentage of cells that stained positively for BrdU for each experimental condition is depicted in the bar graph. $^{**}p<0.01$ *vs.* wild-type LEC (n=6 biologically independent samples for each condition). **b)** Cell migration was quantified after performing a scratch wound in confluent isolated LyECs from wild-type and DHX15$^{+/-}$ that were cultured in 6-well plates. Then images of wound healing were acquired after 0, 7 and 24 hours (n=3 biologically independent samples for each condition). Graph shows the quantification of the wound closure over time expressed as percentage of migration. $^{**}p<0.01$ *vs.* wild-type. All statistical analyses were performed using unpaired two-tailed Student’s t-test. All bar graphs are presented as mean ± SEM.

Supplementary Figure 10. Changes in survival and intratumoral vascular regulators induced by *DHX15* gene deficiency.

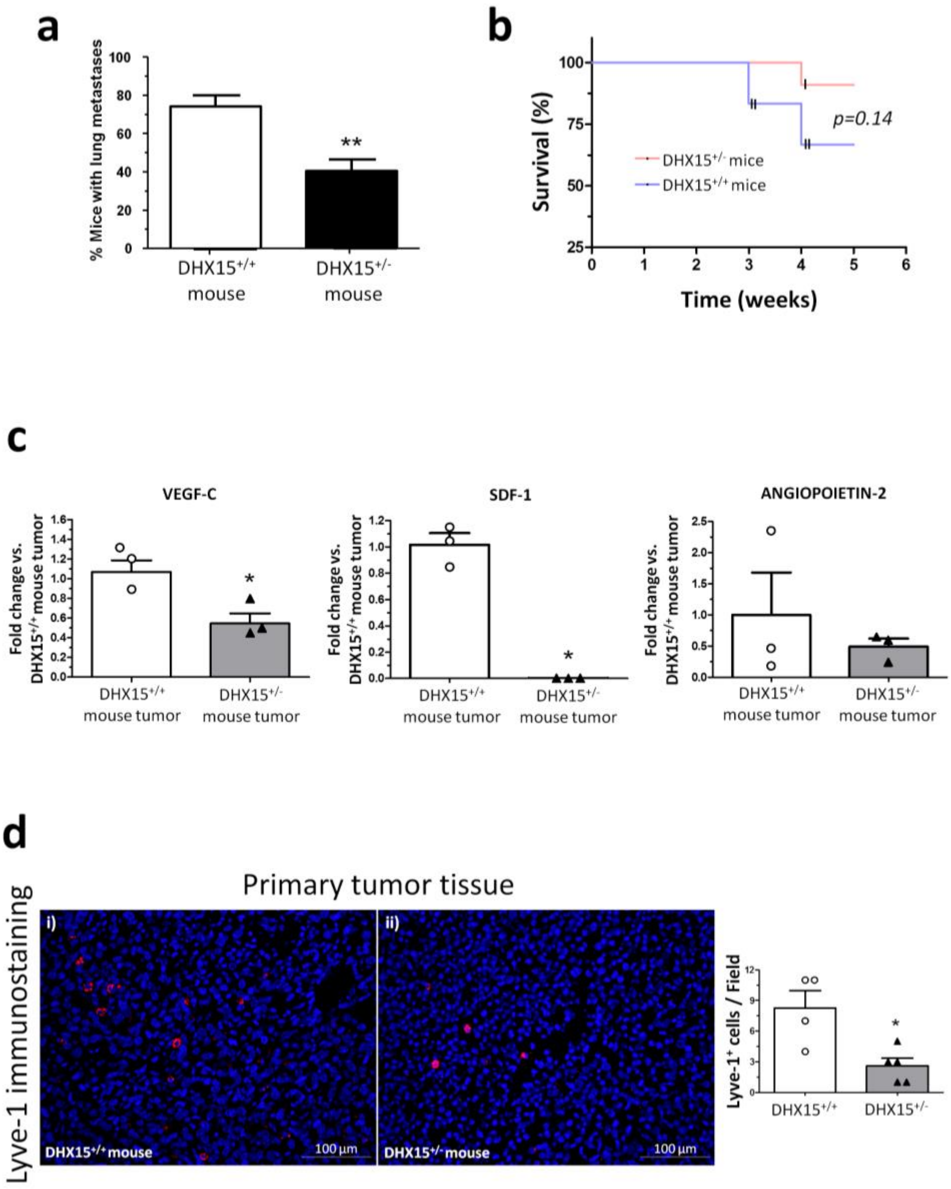

a) Quantification of lung metastases occurrence in wild-type (WT) and DHX15$^{+/-}$ mice. The graph shows the percentage of mice with distant metastases formed in the lungs after primary tumor removal induced by subcutaneous injection of mouse Lewis lung cancer cells (LLC1). ***p*<0.01 *vs*. WT mice (n=15 animals). **b)** Survival graph showing DHX15$^{+/-}$ and DHX15$^{+/+}$ mouse mortality after LLC1 cell implantation. Despite a clear tendency of higher survival in the DHX15$^{+/-}$ group, no significant differences were reached at the end of the experiment (n=15 animals). The vertical tick mark on the staircase graph denote death events for each experimental group. **c)** Primary tumors extracted from wild-type and DHX15$^{+/-}$ mice were lysed in trizol and their mRNA expression was analyzed by real-time PCR, as described in Methods. Graph show the different expression levels for the corresponding genes. mRNA levels are illustrated as fold change relative to *HPRT* mRNA levels. **p*<0.05 *vs*. control (n=3 biologically independent samples for each condition). **d)** Representative Lyve-1 immunostaining in primary tumor extracted from wild-type (panel i) and DHX15$^{+/-}$ (panel ii) mice. Original magnification X200. Graph shows the quantification of Lyve-1 positive cells. **p*<0.05 *vs.* wild-type (n=5 biologically independent samples for each condition). For **a)** statistical analysis was performed using one-side Chi-square test. For **c)** and **d)** statistical analyses were performed using unpaired two-tailed Student's t-test. All bar graphs are presented as mean ± SEM.

Supplementary Figure 11. Uncropped Western blot gels of DHX15, Akt1, Akt2 and GSK-3α/β.

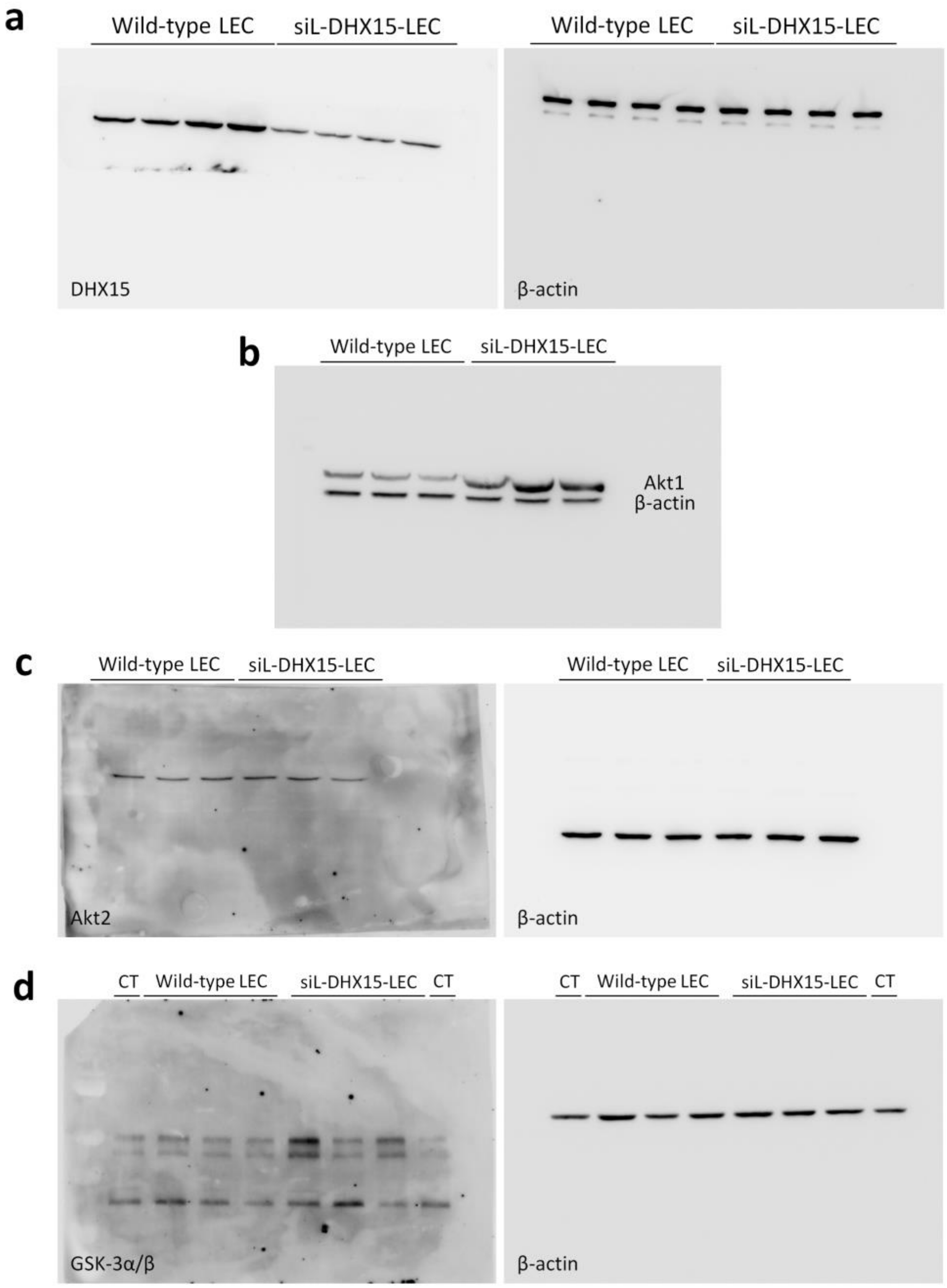

a) DHX15 expression (left) with its corresponding loading control (β-actin, right). **b)** Akt1 expression and its corresponding loading control (β-actin). **c)** Akt2 expression (left) with its corresponding loading control (β-actin, right). **d)** GSK-3α/β expression (left) with its corresponding loading control (β-actin, right). See Figure 1a and b for statistics.

Supplementary Figure 12. Uncropped Western blot gel of Ndufs1 and uncropped gel of Complex I activity.

a

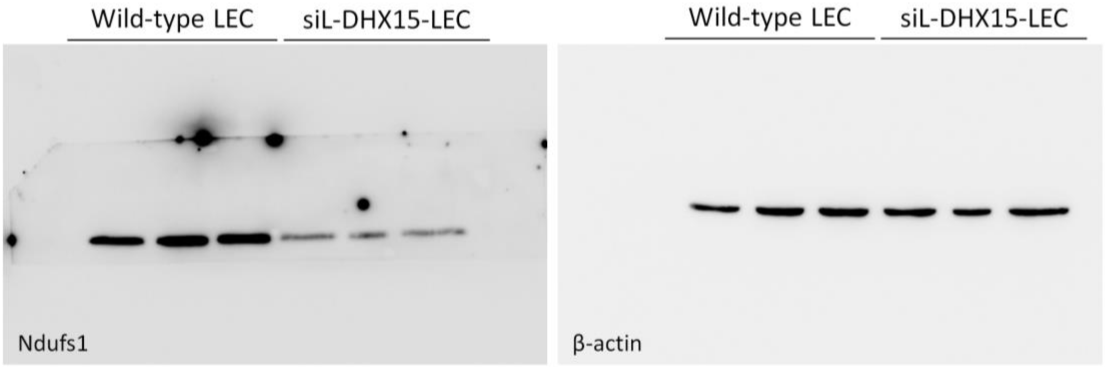

b

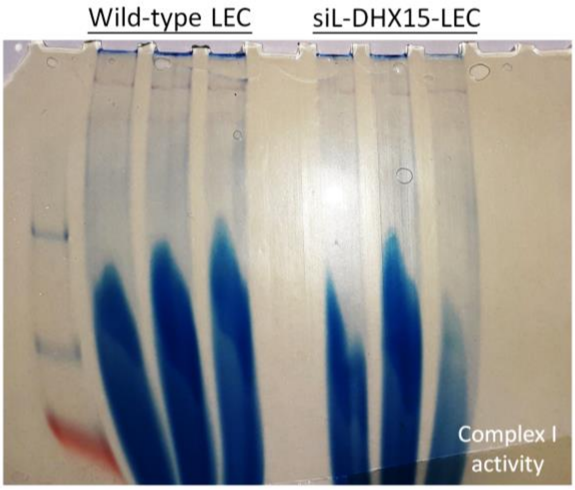

a) Ndufs1 expression (left) with its corresponding loading control (β-actin, right).
b) In-gel complex I activity. See Figure 5a and c for statistics,

Supplementary Figure 13. Uncropped Western blot gels of CD31, eNOS and DHX15.

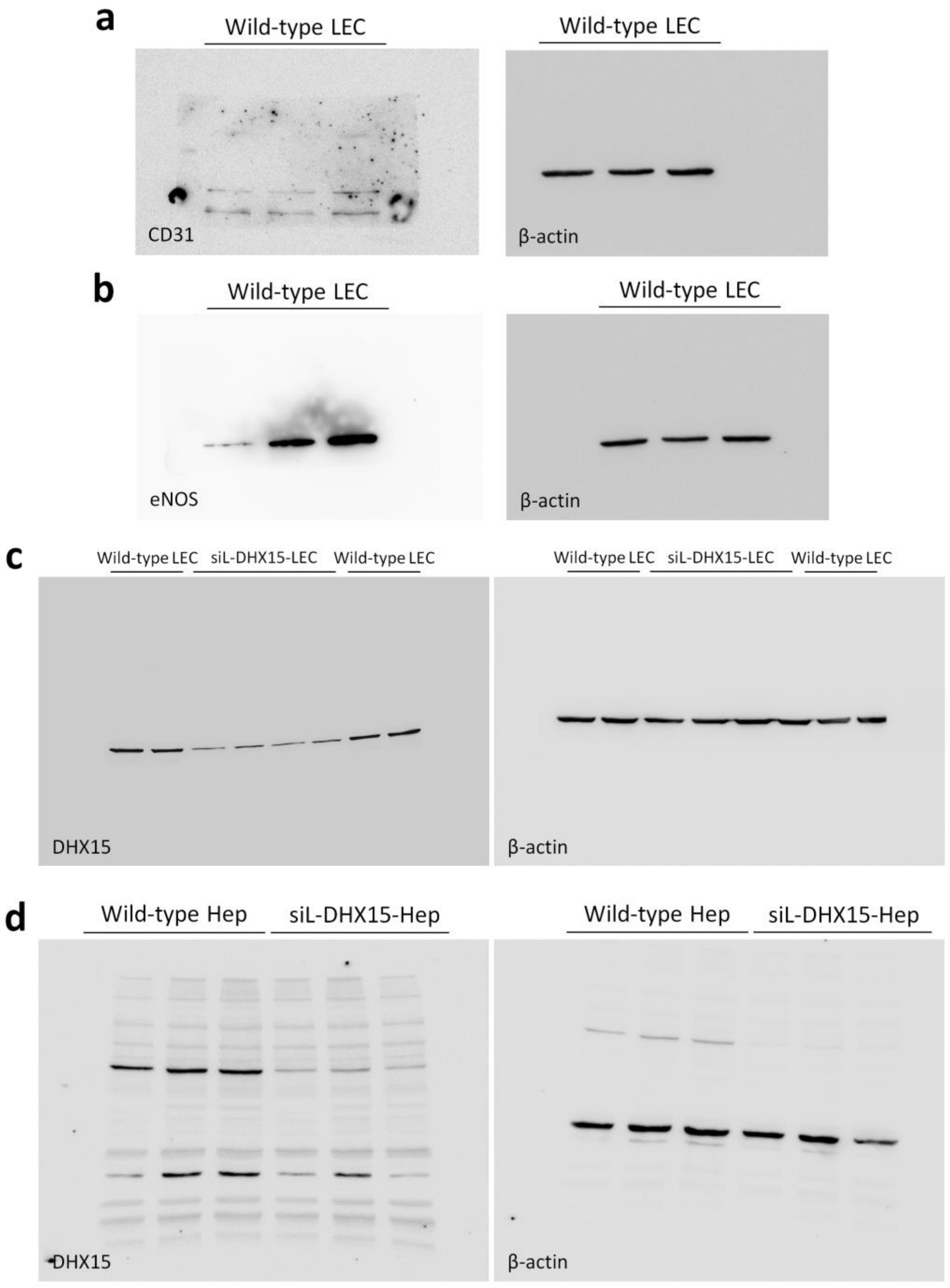

a) CD31 expression (left) with its corresponding loading control (β-actin, right). **b)** eNOS expression and its corresponding loading control (β-actin). **c)** DHX15 expression (left) with its corresponding loading control (β-actin, right). **d)** DHX15 expression (left) with its corresponding loading control (β-actin, right). See Supplementary Figures 1a and b, and 8a for statistics

SUPPLEMENTARY TABLES

Supplementary Table 1. Specific primers for cDNA amplification.

Gene	Species	Forward sequence	Reverse sequence
Podoplanin	Mouse	5'-TGAATCTACTGGCAAGGCACCTCT-3'	5'-TGCTGAGGTGGACAGTTCCTCTAA-3'
Lyve-1	Mouse	5’-AGCAGCATTCAAGAACGAAGCAGC-3’	5’-TTTCACGTAGCAAACAGCCAGCAC-3’
VEGFC	Mouse	5’-CCTGAATCCTGGGAAATGTGCC-3’	5’-CGATTCGCACACGGTCTTCTGT-3’
SDF-1	Mouse	5’-GGAGGATAGATGTGCTCTGGAAC-3’	5'-AGTGAGGATGGAGACCGTGGTG-3’
DHX15	Mouse	5’-CAGAATGGAGCAATTGGAAGA-3’	5’-TGTCAAAGAGGTCTCTGCAATATTAGT-3’
Pyruvate kinase	Mouse	5’-CGAAAAGCCAGTGATGTGGTGG-3’	5’-GATGCCATCGCTCACTTCTAGG-3
G3PDH	Mouse	5'-CATCACTGCCACCCAGAAGACTG-3'	5'-ATGCCAGTGAGCTTCCCGTTCAG-3'
UGGT1	Mouse	5'-GAAGGTGGATGCTCTCCTGTCA-3'	5'-GGTCAACAACGGCTACCACATC-3'
Aldo A	Mouse	5'-TCGCTCCTTAGTCCTTTCGC-3'	5'-GCGATGTCAGACAGCTCCTT-3'
CD31	Mouse	5’-CCAAAGCCAGTAGCATCATGGTC-3’	5'-GGATGGTGAAGTTGGCTACAGG-3’
Ndufs1	Mouse	5’-AGGATATGTTCGCACAACTGG-3’	5’-TCATGGTAACAGAATCGAGGGA-3’
Angiopoietin-2	Mouse	5’-AACTCGCTCCTTCAGAAGCAGC-3’	5’-TTCCGCACAGTCTCTGAAGGTG-3’
HPRT	Mouse	5’-AGTCCCAGCGTCGTGATTAG-3'	5'-TGATGGCCTCCCATCTCCTT-3'
VEGFC	Zebrafish	5’-AAGGGCCCTAACAGAATGTC-3’	5’-TTTGAATGAAGGGTGTCAGG-3’
Actin	Zebrafish	5’-CACAGATCATGTTCGAGACCT-3’	5’-AGGGCGTAACCCTCGTAGAT-3’

Supplementary Table 2. Experimental and commercial details of the antibodies used in the study.

Name	Supplier	Dilution
Anti-Endomucin	Abcam ab106100, lot#GR3270374-7	1:20 or 1:100
Anti-Lyve-1	Abcam ab219556, lot#GR3340292-3	1:100
Anti-DHX15	Abcam ab254591	1:20
Anti-CD31	BD Pharmigen 550274	1:100
Anti-DHX15	Santa cruz sc-271686, lot#D1917	1:1000
Anti-CD31	Cell signaling 3528S, lot#1	1:1000
Anti-β-actin HRP conjugate	Cell signaling 5125S, lot#6	1:1000
Anti- eNOS	BD Bioscience 612707, #lot2146880	1:1000
Anti- Ndufs1	Abcam ab157221, lot#GR117933-8	1:1000
Anti-podoplanin	Sigma P1995, lot#123K4887	1:200
Anti-BrDU Alexa555	BD Biosciences 560210, lot#8241971	1:100
Goat anti-rat Alexa488	Thermo Fisher A11006	1:500
Goat anti-rabbit Alexa488	Thermo Fisher A11008, lot#828814	1:500
Goat anti-mouse Alexa488	Thermo Fisher A11001, lot#745480	1:500
Goat anti-rabbit peroxidase-conjugated	Cell Signaling NA934V, lot#16803301	1:2000
Goat anti-mouse peroxidase-conjugated	Cell Signaling NA931V, lot#17016967	1:2000
