# Supplementary material for: The loss of DHX15 impairs endothelial energy metabolism, lymphatic drainage and tumor metastasis in mice
Source: Commun Biol. 2021 Oct 15;4:1192. doi: 10.1038/s42003-021-02722-w (PMC8519955; doi:10.1038/s42003-021-02722-w)
Supplement: Supplementary file 2 — Description of Additional Supplementary Files [file 42003_2021_2722_MOESM2_ESM.pdf]

Description of Additional Supplementary Files

File name: Supplementary Data 1.
Description: RNA-seq analysis of differentially expressed gene in endothelial cells in response to DHX15 gene silencing.

File name: Supplementary Data 2.
Description: Proteomic analysis of differentially expressed proteins in endothelial cells in response to DHX15 gene silencing.

File name: Supplementary Data 3.
Description: Gene ontology analysis of differentially expressed gene in endothelial cells in response to DHX15 gene silencing.

File name: Supplementary Data 4.
Description: Alternative splicing analysis of gene transcripts in endothelial cells in response to DHX15 gene silencing.

File name: Supplementary Data 5.
Description: Data source of complex I activity kinetic curve.
