# Supplementary material for: The loss of DHX15 impairs endothelial energy metabolism, lymphatic drainage and tumor metastasis in mice
Source: Commun Biol. 2021 Oct 15;4:1192. doi: 10.1038/s42003-021-02722-w (PMC8519955; doi:10.1038/s42003-021-02722-w)
Supplement: Supplementary file 8 — Reporting Summary [file 42003_2021_2722_MOESM8_ESM.pdf]

nature research

Corresponding author(s): Manuel Morales-Ruiz, COMMSBIO-20-3387B

Last updated by author(s): Sep 7, 2021

Reporting Summary

Nature Research wishes to improve the reproducibility of the work that we publish. This form provides structure for consistency and transparency in reporting. For further information on Nature Research policies, see our Editorial Policies and the Editorial Policy Checklist.

Statistics

For all statistical analyses, confirm that the following items are present in the figure legend, table legend, main text, or Methods section.

n/a	Confirmed	
☐	☒	The exact sample size (*n*) for each experimental group/condition, given as a discrete number and unit of measurement
☐	☒	A statement on whether measurements were taken from distinct samples or whether the same sample was measured repeatedly
☐	☒	The statistical test(s) used AND whether they are one- or two-sided<br>*Only common tests should be described solely by name; describe more complex techniques in the Methods section.*
☐	☒	A description of all covariates tested
☐	☒	A description of any assumptions or corrections, such as tests of normality and adjustment for multiple comparisons
☐	☒	A full description of the statistical parameters including central tendency (e.g. means) or other basic estimates (e.g. regression coefficient) AND variation (e.g. standard deviation) or associated estimates of uncertainty (e.g. confidence intervals)
☐	☒	For null hypothesis testing, the test statistic (e.g. *F*, *t*, *r*) with confidence intervals, effect sizes, degrees of freedom and *P* value noted<br>*Give P values as exact values whenever suitable.*
☒	☐	For Bayesian analysis, information on the choice of priors and Markov chain Monte Carlo settings
☐	☒	For hierarchical and complex designs, identification of the appropriate level for tests and full reporting of outcomes
☒	☐	Estimates of effect sizes (e.g. Cohen's *d*, Pearson's *r*), indicating how they were calculated

Our web collection on statistics for biologists contains articles on many of the points above.

Software and code

Policy information about availability of computer code

Data collection	ImageJ version 1.52b; ProteomeDiscoverer software v2.0; Real Time Analysis (RTA) 2.7.6; XFe wave software v2.6
Data analysis	Ingenuity Pathway Software v4.0; DESeq2 version 1.18; RSEM version 1.2.28; STAR version 2.5.2a; R version 3.6 ; Java Treeview software v3.0; Cluster v3.0; GraphPad Prism v5.0

For manuscripts utilizing custom algorithms or software that are central to the research but not yet described in published literature, software must be made available to editors and reviewers. We strongly encourage code deposition in a community repository (e.g. GitHub). See the Nature Research guidelines for submitting code & software for further information.

Data

Policy information about availability of data

All manuscripts must include a data availability statement. This statement should provide the following information, where applicable:

- Accession codes, unique identifiers, or web links for publicly available datasets
- A list of figures that have associated raw data
- A description of any restrictions on data availability

All relevant data are deposited into a public repository and accession codes are provided in the published article

Field-specific reporting

Please select the one below that is the best fit for your research. If you are not sure, read the appropriate sections before making your selection.

☒ Life sciences ☐ Behavioural & social sciences ☐ Ecological, evolutionary & environmental sciences

For a reference copy of the document with all sections, see nature.com/documents/nr-reporting-summary-flat.pdf

Life sciences study design

All studies must disclose on these points even when the disclosure is negative.

Sample size

No sample-size calculation was performed.
Sample sizes were chosen based on: 1 / heuristics: publications showing similar experiments and recommendations from collaborators who are experts for the specific experimental settings performed in our study. 2 / resource constraints: in the case of RNAseq or proteomic experiments. 3 / compliance with The 3Rs and Animal Welfare protocol for the use of animals in scientific research aimed at reducing the number of animals used to a minimum to obtain information from fewer animals.
The rationale for why we are convinced that the sample sizes chosen were sufficient is based on the fact that all the single conclusions stated in our study were based on the results of at least two different experimental methodologies performed to demonstrate the same biological process and each one of these methodologies gave us statistical significance. For example, we demonstrated that DHX15 impaired complex I activity through three different experimental designs: Seahorse experiments, in-gel activity experiments, and colorimetric enzymatic reaction assay.

Data exclusions

No data were excluded from the analyses

Replication

All the replicated data considered for statistical analysis were obtained from independent experiments. All attempts at replication were successful.

Randomization

All the samples and animals were allocated into the different experimental groups randomly.

Blinding

The investigators responsible for data collection were blinded to the real identification of the groups.

Reporting for specific materials, systems and methods

We require information from authors about some types of materials, experimental systems and methods used in many studies. Here, indicate whether each material, system or method listed is relevant to your study. If you are not sure if a list item applies to your research, read the appropriate section before selecting a response.

Materials & experimental systems

n/a	Involved in the study	
☐	☒	Antibodies
☐	☒	Eukaryotic cell lines
☒	☐	Palaeontology and archaeology
☐	☒	Animals and other organisms
☒	☐	Human research participants
☒	☐	Clinical data
☒	☐	Dual use research of concern

Methods

n/a	Involved in the study	
☒	☐	ChIP-seq
☐	☒	Flow cytometry
☒	☐	MRI-based neuroimaging

Antibodies

Antibodies used

Anti-Endomucin (Abcam ab106100, lot#GR3270374-7), used at 1:20 or 1:100
Anti-Lyve-1 (Abcam ab219556, lot#GR3340292-3), used at 1:100
Anti-DHX15 (Abcam ab254591), used at 1:20
Anti-CD31 (BD Pharmigen 550274), used at 1:100
Anti-DHX15 (Santa cruz sc-271686, lot#D1917) used at 1:1000
Anti-CD31 (Cell signaling 3528S, lot#1) used at 1:1000
Anti-β-actin HRP conjugate (Cell signaling 5125S, lot#6) used at 1:1000
Anti- eNOS (BD Bioscience 612707, #lot2146880) used at 1:1000
Anti- Ndufs1 (Abcam ab157221, lot#GR117933-8) used at 1:1000
Anti-podoplanin (Sigma P1995, lot#123K4887), used at 1:200
Anti-BrDU Alexa555 (BD Biosciences 560210, lot#8241971), used at 1:100
Goat anti-rat Alexa488 (Thermo Fisher A11006) used at 1:500 dilution
Goat anti-rabbit Alexa488 (Thermo Fisher A11008, lot#828814) used at 1:500 dilution
Goat anti-mouse Alexa488 (Thermo Fisher A11001, lot#745480) used at 1:500 dilution

	Goat anti-rabbit peroxidase-conjugated (Cell Signaling NA934V, lot#16803301) used at 1:2000 dilution<br>Goat anti-mouse peroxidase-conjugated (Cell Signaling NA931V, lot#17016967) used at 1:2000 dilution
Validation	Anti-Endomucin (Abcam) has been validated in ICC/IF and tested in mouse by Abcam.<br>Anti-Lyve-1 (Abcam) was validated in WB, IP, IHC and tested in Human by Abcam.<br>Anti-DHX15 (Abcam) was validated in WB, IHC, ICC/IF and tested in Mouse, Rat, Human by Abcam.<br>Anti-CD31 (BD Pharmigen) was tested for IHC in mouse spleen, lung, heart, and thymus by BD Pharmigen.<br>Anti-DHX15 (Santa Cruz) was validated in mouse, rat and human origin by WB, IP, IF, IHC(P) and ELISA by Santa Cruz.<br>Anti-CD31 (Cell Signaling) was validated in WB, IP, IHC, IF in mouse by Cell Signaling.<br>Anti-β-actin HRP conjugate (Cell signaling) was validated in WB by Cell Signaling.<br>Anti- eNOS (BD Bioscience) was validated in WB by BD Bioscience<br>Anti- Ndufs1 (Abcam) has been validated in Flow Cytometry, WB, ICC/IF, IHC-P in human by Abcam.<br>Anti-podoplanin (Sigma) has been validated in IHC and WB by Sigma.<br>Anti-BrDU Alexa555 (BD Biosciences) has been tested for bioimaging by BD biosciences.<br>Goat anti-rat Alexa488, Goat anti-rabbit Alexa488 and Goat anti-mouse Alexa488 (Thermo Fisher) have been validated in ICC/IF by Thermo Fisher.<br>Goat anti-rabbit peroxidase-conjugated and Goat anti-mouse peroxidase-conjugated (Cell Signaling) have been validated in Wb by Cell Signaling.

Eukaryotic cell lines

Policy information about cell lines

Cell line source(s)	Mouse primary hepatic endothelial cells and mouse primary hepatocytes both immortalized with the SV40 virus (LEC and Hep) were obtained from abmGood. DHX15 silencing was achieved through shRNA by lentiviral infection (Dharmacon).
Authentication	Cell lines used were not authenticated
Mycoplasma contamination	All the cell lines tested negative for mycoplasma contamination
Commonly misidentified lines<br>(See ICLAC register)	No commonly misidentified lines were used

Animals and other organisms

Policy information about studies involving animals; ARRIVE guidelines recommended for reporting animal research

Laboratory animals	mus musculus, C57/BL6, male and female, age 3, 4, 6 or 8 months old mice were used for the animal experiments.<br>danio rerio, male and female, from 24 hours post fertilization until 10 days post fertilization were used for the zebrafish studies.
Wild animals	The study did not involve wild animals
Field-collected samples	The study did not involve samples collected from the field
Ethics oversight	The Investigation and Ethics Committees of the Hospital Clínic and the University of Barcelona

Note that full information on the approval of the study protocol must also be provided in the manuscript.

Flow Cytometry

Plots

Confirm that:

☒ The axis labels state the marker and fluorochrome used (e.g. CD4-FITC).

☒ The axis scales are clearly visible. Include numbers along axes only for bottom left plot of group (a 'group' is an analysis of identical markers).

☒ All plots are contour plots with outliers or pseudocolor plots.

☒ A numerical value for number of cells or percentage (with statistics) is provided.

Methodology

Sample preparation	As described in the supplemental material section, when immortalized hepatic endothelial cells reached about 60-70% of confluence, BrdU (10 mM) was added to the culture medium for 1h. Then, the BrdU-labeled cells were fixed and the DNA was denatured in fixative solution for 1h at 37°C. Next, the cells were incubated with Alexa Fluor 555-conjugated anti-BrdU antibody for 1h at room temperature. Immunofluorescence was detected by flow cytometry (LSRFortessa).
Instrument	LSR Fortessa was used for sorting and analysis.
Software	Data were analyzed in FACSDiva Version 6.1.3.

Cell population abundance	N/A
Gating strategy	1.) SSC vs. FSC gating to exclude debris. 2.) FSC-H vs. FSC-A gating to exclude doublets.<br>3.) FSC-A vs. Alexa 555 (BrDU) gating to quantify BrDU+ cells.

☒ Tick this box to confirm that a figure exemplifying the gating strategy is provided in the Supplementary Information.
